# LAMC1 upregulation via TGFβ induces inflammatory cancer‐associated fibroblasts in esophageal squamous cell carcinoma via NF‐κB–CXCL1–STAT3

**DOI:** 10.1002/1878-0261.13053

**Published:** 2021-07-22

**Authors:** Lingling Fang, Yun Che, Chaoqi Zhang, Jianbing Huang, Yuanyuan Lei, Zhiliang Lu, Nan Sun, Jie He

**Affiliations:** ^1^ Department of Thoracic Surgery National Cancer Center/National Clinical Research Center for Cancer/Cancer Hospital Chinese Academy of Medical Sciences and Peking Union Medical College Beijing China

**Keywords:** CAF, CXCL1, ESCC, heterogeneity, LAMC1, transforming growth factor β

## Abstract

Cancer‐associated fibroblasts (CAF) are a heterogeneous cell population within the tumor microenvironment,and play an important role in tumor development. By regulating the heterogeneity of CAF, transforming growth factor β (TGFβ) influences tumor development. Here, we explored oncogenes regulated by TGFβ1 that are also involved in signaling pathways and interactions within the tumor microenvironment. We analyzed sequencing data of The Cancer Genome Atlas (TCGA) and our own previously established RNA microarray data (GSE53625), as well as esophageal squamous cell carcinoma (ESCC) cell lines with or without TGFβ1 stimulation. We then focused on laminin subunit gamma 1 (LAMC1), which was overexpressed in ESCC cells, affecting patient prognosis, which could be upregulated by TGFβ1 through the synergistic activation of SMAD family member 4 (SMAD4) and SP1. LAMC1 directly promoted the proliferation and migration of tumor cells, mainly via Akt–NFκB–MMP9/14 signaling. Additionally, LAMC1 promoted CXCL1 secretion, which stimulated the formation of inflammatory CAF (iCAF) through CXCR2–pSTAT3. Inflammatory CAF promoted tumor progression. In summary, we identified the dual mechanism by which the upregulation of LAMC1 by TGFβ in tumor cells not only promotes ESCC proliferation and migration, but also indirectly induces carcinogenesis by stimulating CXCL1 secretion to promote the formation of iCAF. This finding suggests that LAMC1 could be a potential therapeutic target and prognostic marker for ESCC.

## Graphical abstract

Inflammatory cancer‐associated fibroblasts (iCAF) are known to promote tumor progression. In our study we investigated the role of TGFβ1 on iCAF formation in esophageal squamous cell carcinoma (ESCC). TGFβ1 upregulated LAMC1 expression by activating SMAD4/SP1, which was abrogated by a TGFβR1 inhibitor (SB505124). The proliferation and migration of tumor cells was promoted by LAMC1 via the Akt/NF‐κB/MMP9‐MMP14 pathway. Additionally, LAMC1 facilitated CXCL1 secretion by activating NF‐κB. Tumor‐secreted CXCL1 stimulated the formation of iCAF through CXCR2/pSTAT3, which was blocked by a CXCR2 inhibitor (SB225002). In summary, iCAF promoted ESCC progression by secreting pro‐inflammatory cytokines and chemokines.

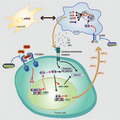

**Abbreviations**
APC: allophycocyanin
CAF: cancer‐associated fibroblasts
ChIP: chromatin immunoprecipitation
CM: conditioned medium
ECM: extracellular matrix
ESCC: esophageal squamous cell carcinoma
GSEA: gene set enrichment analysis
iCAF: inflammatory CAF
LAMC1: laminin subunit gamma 1
MIF: macrophage migration inhibitory factor
MMP: matrix metalloproteinase
myCAF: myofibroblasts
rCXCL1: recombinant CXCL1
RT‐qPCR: quantitative real time PCR
shRNA: short hairpin RNA ()
SMAD4: SMAD family member 4
TCGA: The Cancer Genome Atlas
TGFβ: transforming growth factor β
TME: tumor microenvironment
TNFα: tumor necrosis factor α

## 1 Introduction

Esophageal cancer is one of the most common cancers, 90% of which is esophageal squamous cell carcinoma (ESCC) [1]. ESCC is highly malignant, with a 5‐year survival rate between 15% and 25% [2]. The tumor microenvironment (TME) is mainly composed of immune cells, fibroblasts, endothelial cells and extracellular matrix (ECM), which play an important role in promoting tumor cell proliferation, inhibiting tumor cell apoptosis and causing immune escape [3, 4, 5]. As an important member of the TME, cancer‐associated fibroblasts (CAF) mainly secrete cytokines, modify the ECM, and interact with tumor cells and other cells [6, 7, 8]. For example, CAF can secrete VEGF to promote tumor cell angiogenesis [9], and CAF‐secreted interleukin (IL)6 and FAP increase proliferation and tumor recurrence [10, 11, 12, 13]. CAF have been used as a therapeutic target in many previous studies but the results were disappointing [14, 15, 16], which may be due to the heterogeneity of CAF [17, 18]. Similar to tumor cells, different subgroups of CAF exist among different tumors and within the same tumor [19, 20, 21]. According to the different functions of CAF in tumors, CAF are divided into tumor‐promoting CAF, tumor‐suppressing CAF and neutral CAF in gastrointestinal tumors [20]. In pancreatic ductal adenocarcinoma, CAF are classified into distinct inflammatory CAF (iCAF) and myofibroblasts (myCAF), which differ not only in their transcriptional profile but also significantly in their tumor distribution location and effect on tumors [17, 19]. In breast cancer, the specific subgroup of CD10^+^ and GPR77^+^ CAF can maintain the stemness of tumor cells by secreting IL6 and IL8 and causing chemotherapy resistance. Targeting this specific CAF subgroup can reverse chemotherapy resistance in breast cancer [22]. The heterogeneity of CAF is influenced by paracrine and other pathways in other cells, such as IL1‐induced LIF expression and downstream JAK/STAT activation to generate iCAF [19, 23].

The regulation of CAF heterogeneity by transforming growth factor β (TGFβ) is controversial. The TGFβ signaling pathway has both oncogenic and anticancer effects in ESCC [3, 24, 25]. In the early stage, tumor growth is inhibited by reduced TGFβ responsiveness. However, it later also promoted tumor invasion and metastasis. This ‘switch’ in carcinogenesis may be related to the absence of adaptor proteins, such as β2‐spectrin [3]. This contradictory effect of TGFβ not only exists in tumor cells but also regulates the phenotype of CAF. The exosomes secreted by tumor cells contain TGFβ and induce the transformation of fibroblasts into activated CAF in bladder cancer [23]. In prostate cancer, bone marrow‐derived mesenchymal stem cells secrete TGFβ, which promotes the conversion of normal fibroblasts into CAF with tumorigenicity [26]. However, tumor cell‐secreted TGFβ antagonizes the activation of IL1 signaling and inhibits the transformation of myCAF into iCAF, which plays an anticancer role in pancreatic ductal adenocarcinoma [19].

Overall, the tumorigenic effect of TGFβ on tumor cells has a dual role: it can affect tumor cells directly and indirectly through TME cell cross‐talk, especially between tumor cells and CAF [27]. We explored oncogenes regulated by TGFβ1, which are involved in signaling molecules and interactions in the TME. Through bioinformatics analysis, we hypothesized that laminin subunit gamma 1 (LAMC1) is an oncogene that is upregulated by TGFβ1 and participates in cell‐to‐cell signal transduction in the TME in ESCC. Many previous studies have shown that high LAMC1 expression promotes tumor progression and can be used as a prognostic biomarker in many cancers, such as hepatocellular carcinoma, colorectal cancer and endometrial cancer [28, 29]. LAMC1 is involved in many carcinogenic effects within tumor cells. For example, miR‐29b‐3p negatively regulates LAMC1 to inhibit melanoma invasion [30]. LAMC1 can also promote the Warburg effect by upregulating PKM2 in hepatocellular carcinoma [31]. This study explored the carcinogenic effect of LAMC1 in ESCC from two aspects: the direct effect on tumor cells and indirect effect through the TME, especially CAF. We hypothesized that high expression of LAMC1, upregulated by TGFβ1, affected the prognosis of ESCC patients, and LAMC1 was involved in signaling molecules and interaction pathways in the TME. This study focused on exploring the tumorigenic mechanism of LAMC1 and its role in the microenvironment, especially in the interaction of tumor cells and CAF.

## 2 Materials and methods

### 2.1 Patients and tumor samples

A paraffin‐embedded ESCC microarray containing 55 ESCC tissues and 50 adjacent tissues was purchased from Outdo Biotech (catalog no. HEsoS105Su01, Shanghai, China) for immunohistochemistry; 12 fresh ESCC tissues were obtained at our hospital for isolation of CAF in 2018. All patients signed an informed consent. Our study was approved by the Committee for the Ethics Review of Research Involving Human Subjects of the Cancer Hospital of the Chinese Academy of Medical Sciences. Our study methodologies conformed to the standards set by the Declaration of Helsinki.

### 2.2 Immunohistochemistry and scoring

Immunohistochemistry (IHC) was performed as previously described [32]. The difference is that we used anti‐LAMC1 antibody (diluted 1 : 200, catalog no. AP20488PU‐N, OriGene, Rockville, MD, USA). The final immunoreactivity score (IRS) was the product of staining intensity and percentage of positive cells.

### 2.3 Cell culture and stable cell construction

Human ESCC cell lines KYSE30 and KYSE450 were cultured as in a previous study [33]. Medical Research Council cell strain‐5 (MRC‐5; KG508, Keygene, Nanjing, China) was cultured in MEM (minimum Eagle’s medium), with 10% FBS, 1% nonessential amino acid and 1 mm sodium pyruvate. Two short hairpin RNA (shRNA) oligonucleotides (5’‐GCAAGTTCTGACAGGACTACC‐3’ and 5’‐GCAAGACAGTGGTTCTTATGA‐3’) were inserted into pLV‐Puro for knockdown SP1 (SyngenTech, Beijing, China), two shRNA oligonucleotides (5’‐GCCAGCTACTTACCATCATAA‐3’ and 5’‐GCTCCTAGACGAAGTACTTCA‐3’) were inserted into pLV‐neo for knockdown SMAD4 (SyngenTech) and two shRNA oligonucleotides (5’‐GCTGGTGTGTAATTGCAAACACGAATGTTTGCAATTACACACCAGC‐3’ and 5’‐GCTACTTTCCTCGGTACTTCACGAATGAAGTACCGAGGAAAGTAGC‐3’) were inserted into pLV‐Puro (SyngenTech) for knockdown LAMC1. The negative control (NC) sequence was 5’‐AAACGTGACACGTTCGGAGAACGAATTCTCCGAACGTGTCACGTTT‐3’ (SyngenTech). Lenti ORF clone of human LAMC1 and vector was purchased form OriGene (RC216928L4, PS100093) for overexpression of LAMC1. As previous descried, we then constructed knockdown SP1, knockdown SMAD4, knockdown SP1 combined with SMAD4 and knockdown LAMC1 or overexpression of LAMC1 ESCC cells and the controls. The expression of LAMC1, SP1 and SMAD4 in the infected cells was confirmed by quantitative (RT‐qPCR) and western blot, 96 h after infection.

### 2.4 Isolation of CAF and coculture system

As described in our previous study, homogeneous CAF was isolated from fresh tumor tissue and identified using the cellular immunofluorescence marker αSMA [33]. Homogeneous CAF were obtained for further analysis. All CAF used in the experiment were maintained for no more than 10 passages. For the coculture system, CAF were seeded in a 6‐well or 24‐well plate with or without SB225002 in the medium, and the ESCC cells were placed in the upper chamber with a 0.4‐μm pore size (Corning, 3412, 3413, New York, NY, USA). CAF and ESCC cells were cocultured at a 1 : 1 ratio; approximately 2 × 10^5^/well CAF or ESCC cells were used for 6‐well plates, and 3 × 10^4^/well CAF or ESCC cells were used for 24‐well plates. Cells were cocultured for 48 h, and the RNA and proteins of CAF extracted.

### 2.5 Immunofluorescence staining

For cell staining, α‐smooth muscle actin (diluted 1 : 100, Abcam, Cambridge, UK) was used to verify CAF as previously described [33]. The UltraVIEW VoX high‐speed laser confocal real‐time imaging analysis system for living cells (Olympus, Tokyo, Japan) was used.

### 2.6 Collection of conditioned medium (CM)

As previously described, CM was collected after cells had been cultured in serum‐free medium for 24 h and was centrifuged at 1000 ***g*** for 5 min [33]. We collected CM of stable knockdown and overexpression of LAMC1 KYSE30 and KYSE450 cells with or without different treatments [PBS, tumor necrosis factor α (TNF α), MK‐2206, JSH], the CM of CAF cocultured with shLAMC1 and sh‐vector ESCC cells with or without SB225002, and CAF with different intervention conditions (PBS, CM of shLAMC1, recombinant CXCL1, SB225002). The CM was concentrated only 40‐fold for western blotting via a Centricon Centrifugal filter (3 kd, Millipore, Temecula, CA, USA). The CM used to stimulate cells was sterile filtered and diluted once with medium.

### 2.7 Drugs

For the treatment of KYSE30 and KYSE450 cells, recombinant TGFβ1 (R&D, Minneapolis, MN, USA) was used at a final concentration of 10 ng·mL^−1^ unless otherwise specified. TNFα (PeproTech, Suzhou, China) was used at a final concentration of 10 ng·mL^−1^. Treatment periods were 24 h unless otherwise specified. We used 10 μm SB505124 and 10 μm JSH‐23 (Selleck, Houston, TX, USA) to inhibit TGFβ signaling and NF‐κB signaling, respectively. These inhibitors were administered to the cells 30 min before any other treatments. In addition, 5 µm MK‐2206 2HCI (Selleck) was used to inhibit Akt phosphorylation selectively for 24 h. CAF were treated with 10 ng·mL^−1^ recombinant CXCL1 (PeproTech) for 24 h. SB225002 (Selleck) was added 1 h prior to inhibiting CXCL1/CXCR2.

### 2.8 RNA extraction and quantitative RT‐qPCR

The total RNA was extracted from the cultured cells using a standard TRlzol protocol (Thermo Fisher Scientific, Pittsburgh, PA, USA). Complementary DNA (cDNA) was synthesized using a RevertAid First‐Strand cDNA Synthesis Kit (Thermo Fisher Scientific) and 1 μg RNA was used for cDNA synthesis. RT‐qPCR was performed on an ABI 7900HT Real‐Time PCR Thermocycler (ABI, Pittsburgh, PA, USA). The$2^{‐\Delta \Delta C_{t}}$ method was used to quantify the relative RNA expression level, and β‐actin was used as an endogenous reference. At least two independent experiments were conducted with a minimum of three technical replicates per experiment. All primers and oligonucleotides used in this study are listed in Supporting Information Table S4.

### 2.9 Western blotting

Unless specific, the proteins isolated from the cultured cells were prepared using RIPA buffer supplemented with a protease and phosphatase inhibitor cocktail, and CM were concentrated with Amicon Ultra‐15 Centrifugal Filter Devices (Millipore). All the proteins were quantified with a BCA protein assay kit (Thermo Fisher Scientific). Western blot was performed as previously described [33]. All antibodies used for western blotting are listed in Supporting Information Table S5.

### 2.10 RNA sequencing

LAMC1 knockdown and control of KYSE30 and KYSE450 cells were used to perform mRNA sequencing (mRNA‐seq) with Illumina HiSeq 4000. In another experiment, CAF with PBS, rCXCL1 or CM of shLAMC1 was also used to perform the sequencing. The mRNA‐seq was performed by Boao (Beijing, China).

### 2.11 Cell proliferation and apoptosis induction assay

CCK8 assays (KeyGEN) were performed following the manufacturer's instructions. For CCK8 assays, 2000/well KYSE30 cells, 2500/well KYSE450 cells and 2000/well CAF were seeded in 96‐well plates. The ESCC cells were seeded into 6‐well plates, cultured for 24 h and then treated with cisplatin (Sigma‐Aldrich) (10 μm) for 24 h. Using an apoptosis detection kit (KeyGEN), cells were double‐labeled with Annexin V–allophycocyanin (APC) and PI according to the manufacturer's instructions, using 5 μL Annexin V‐APC and 5 μL PI/1 × 10^6^ cells.

### 2.12 Boyden chamber transwell assays

We used 24‐well Boyden chambers precoated without Matrigel matrix (Corning) for migration assays, respectively. Transfected or wild‐type (WT) cells (1 × 10^4^ KYSE30 or 5 × 10^4^ KYSE450) were plated into the upper chamber and cultured in serum‐free RPMI 1640 medium with stimuli or PBS. The cells were then allowed to translocate toward medium containing 20% FBS for 24 h. Cells on the lower side of the chamber were fixed and stained with 1% crystal violet. We then counted the numbers of those cells in five different areas at 100‐fold magnification.

### 2.13 Luminex liquid suspension chip detection

We selected CM of shLAMC1 KYSE450 cells and the controls. Each sample was duplicated. Luminex liquid suspension chip detection was performed by Wayen Biotechnologies (Shanghai, China). The Bio‐Plex Pro Human Chemokine Panel 48‐plex kit was used according to the manufacturer’s instructions. Each CM was incubated in 96‐well plates embedded with microbeads for 1 h, and then incubated with detection antibody for 30 min. Furthermore, streptavidin‐PE was filled in each well for 10 min, and values were read using the Bio‐Plex MAGPIX System (Bio‐Rad, Hercules, CA, USA).

### 2.14 Enzyme‐linked immunosorbent assay (ELISA)

A 100‐mL aliquot of CM from each well of cultured cells was collected at 48 h for measurement of CXCL1 protein (without dilution) with a Human GRO‐alpha ELISA kit (ELH‐GROa, RayBio, Guangzhou, China). Each sample had duplicate wells for the standard curve and experimental samples. The concentration was calculated according to the manufacturer’s instructions.

### 2.15 Chromatin immunoprecipitation assay (ChIP)

KYSE30 cells were stimulated by recombinant TGFβ1 at 5 ng·mL^−1^ for 30 min, and the cells then used to perform chromatin immunoprecipitation assay (ChIP) with an anti‐Smad4 (catalog no. 46535, CST, Danvers, MA, USA) or SP1 (catalog no. 9389, CST) antibody (catalog no. 9389, CST) and Enzymatic Chromatin IP kit (catalog no. 9003, CST) according to the manufacturer’s instructions. The purified DNA was analyzed by RT‐qPCR and PCR with specific primers for the promoter area of LAMC1 (Table S2). Furthermore, before DNA purification, chromatin fractions mixed with magnetic beads were prepared in 1× SDS buffer for western blot analysis.

### 2.16 Tumor xenograft experiments

All animal experiments were approved by the Institutional Animal Care and Use Committee of the Cancer Hospital, Chinese Academy of Medical Sciences and Peking Union Medical College. Feeding conditions are as follows: a feeding room free of special pathogenic bacteria with dust removal and bacteria removal, temperature controlled between 25 and 27 °C, humidity controlled between 45 and 50%, and fresh filtered air. As previously described, 1 × 10^6^ LAMC1 knockdown or overexpression of KYSE30 cells and control cells were subcutaneously injected into the flanks of BALB/cA‐nu nude mice (Beijing HFK Bio‐Technology, China) to establish tumor xenografts (six mice per group) [32, 34]. In another experiment, MRC‐5 cells were stimulated with 20 ng·mL^−1^ TGFβ1 for 4–5 days before further animal experiments. A total of 1 × 10^6^ WT KYSE30 cells alone or 5 × 10^5^ WT KYSE30 cells admixed with 5 × 10^5^ MRC‐5 cells (or rCXCL1‐pretreated MRC‐5 cells) were resuspended in 0.2 mL of PBS and then subcutaneously injected into the flanks of mice to establish tumor xenografts (six mice per group). Subgroups of mice were treated with SB225002 (1 mg·kg^−1^) intraperitoneally once every 2 days until the tumors reached 5 mm in diameter. The tumor volume was calculated using the formula *V* = (*L*◊*W* ^2^)/2. All BALB/c nude mice were sacrificed 3–4 weeks later, and the tumors excised and weighed.

### 2.17 Lung colonization assay

As described previously, cells were injected into female NOD‐SCID mice (Beijing HFK Bio‐Technology) through the tail vein. A total of 1 × 10^6^ sh‐1, sh‐vec, LAMC1 and vector KYSE30 cells were injected (six mice per group) [34]. The mice were sacrificed 7 weeks later, and the lungs were excised and fixed with 4% polysorbate, followed by embedding in paraffin for hematoxylin and eosin (H&E) staining. The number of lung surface metastatic nodes was calculated by gross and microscopic examination as previously described [34].

### 2.18 Statistical analysis

prism graphpad (version 6.0, GraphPad Software Inc., San Diego, CA, USA), SPSS, gene set enrichment analysis (GSEA) and R script were used. Correlations between mRNA expression levels were analyzed using Pearson’s correlation coefficient. A chi‐square test was performed to determine the relationship between clinicopathological variables and LAMC1 expression. Overall survival (OS) curves were analyzed using the Kaplan–Meier method and log‐rank tests. The differences between groups were analyzed using a two‐tailed *t*‐test. The data are presented as the mean ± standard deviation (SD). Differences were considered significant at *P* < 0.05 and are indicated as ****P < 0.0001, ***P < 0.001, **P < 0.01, and *P < 0.05 (ns, not significant).

## 3 Results

### 3.1 LAMC1‐was upregulated by TGFβ through the synergistic activation of SMAD4 and SP1 and predicted a poor prognosis in ESCC

**Fig. 1:**
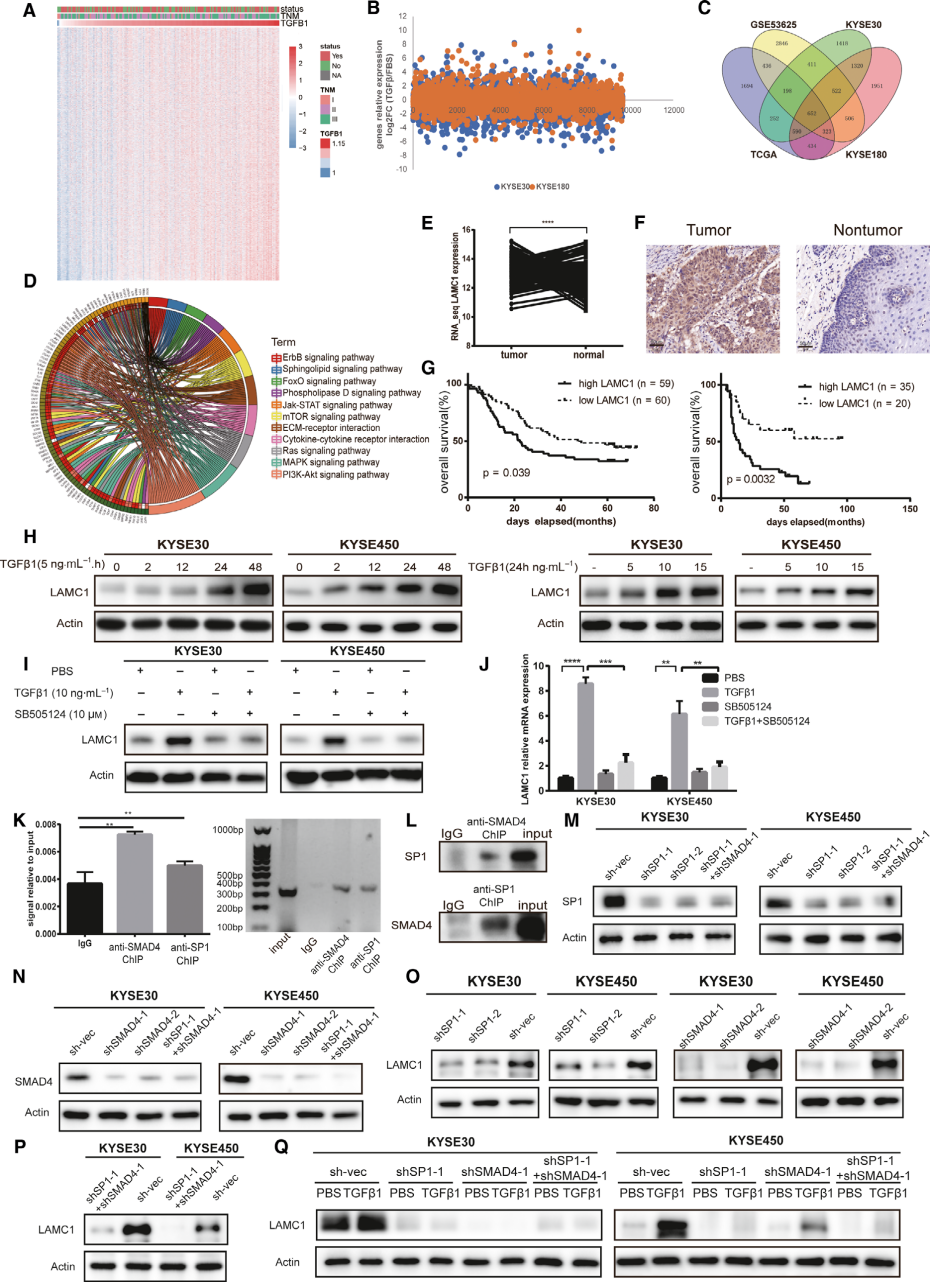
Laminin subunit gamma 1 (LAMC1) expression was upregulated by transforming growth factor β (TGFβ1) via SMAD family member 4 (SMAD4) and SP1 synergistic activation and was associated with a poor prognosis in patients with esophageal squamous cell carcinoma (ESCC). (A) Heatmap of the RNA‐seq data listing genes positively correlated with TGFβ1 in ESCC of GSE53625 (*r* > 0.15, *P* < 0.05). (B) A total of 3084 genes of KYSE30 and KYSE180 were upregulated after TGFβ1 treatment using RNA‐seq data (fold change > 1). (C) Venn diagram of 652 overlapping genes based on positive regulation by TGFβ1 in ESCC. (D) The 31 genes of 652 genes were enriched in 11 pathways involved in the tumor microenvironment. (E,F) LAMC1 was more highly expressed in cancer than in para‐cancer at the RNA and protein levels. Representative immunohistochemical (IHC) images of LAMC1 staining in ESCC tumor tissues and nontumor tissues (original magnification: 200x). (G) Kaplan–Meier survival analysis of OS based on high (*n* = 89) and low (*n* = 90) LAMC1 expression in GSE53625 (left) and based on high (*n* = 35) and low (*n* = 20) LAMC1 expression (by IHC, right). (H,I) As verified by western blot and RT‐qPCR, TGFβ1 upregulated LAMC1 expression in a concentration‐ (at different concentrations for 24 h) and time‐dependent manner (at 5 ng·mL^−1^ for different times) in KYSE30 and KYSE450 cells (H), which could be reversed by the TGFβR1 inhibitor SB505124 (10 μm) at the protein (I) and RNA (J) levels. (K,L) After KYSE30 cells were treated with TGFβ1 (5 ng·mL^−1^) for 30 min, the localization of SMAD4 or SP1 to the LAMC1 promoter was detected by ChIP, and the expression of SMAD4 or SP1 was detected in anti‐SP1 or anti‐SMAD4 chromatin fractions separately by western blot (L). (M,N) Knockdown efficiency of SP1 combined with or without SMAD4 knockdown in ESCC cells was verified by western blotting. (O–Q) The expression levels of LAMC1 decreased in ESCC cells with SP1 knockdown combined with (P) or without (O) knockdown, but this effect could not be rescued by TGFβ1 treatment (Q). Three biological replicates were performed for *in vitro* assays. The data in bar charts are presented as the mean ± SD. **P < 0.01, ***P < 0.001, ****P < 0.0001 (Student’s *t*‐test).

Previous research has established that TGFβ plays an important role in the TME, especially in cell‐to‐cell signaling [3]. We sought to identify genes regulated by TGFβ1; those genes were found to be involved in signaling molecules and interaction pathways of the TME in ESCC. Furthermore, the genes themselves could influence the prognosis of patients with ESCC. We made these observations through the following process. First, through analysis of our mRNA microarray data (GSE53625), we found that 4130 genes were upregulated in cancer tissues compared with adjacent tissues (Log2FC > 0.5, false discovery rate (FDR) < 0.001) and 238 genes were significantly (*P* < 0.05) associated with poor prognosis. Secondly, using Pearson's correlation analysis of GSE53625 and TCGA data, we found that 1609 genes were positively correlated with TGFβ1 expression in ESCC cancer tissues (*r* > 0.15, FDR < 0.05) (Figs 1A and S1A). At the same time, in our previous study where RNA‐seq was performed on TGFβ1‐treated and untreated ESCC cells [34], we found that a total of 3084 genes were upregulated (Log2FC > 0, FDR < 0.05) (Fig. 1B). Combining the two results, we found that a total of 625 genes were co‐expressed with TGFβ1 and were upregulated by TGFβ1 (Fig. 1C). Enrichment analysis of these genes (KOBAS) [35] (FDR < 0.0001) revealed that 11 pathways were involved in environmental information processing, whereas only two pathways, cytokine–cytokine receptor interaction and ECM receptor interaction, were included in signaling molecules and interaction pathways (Figs 1D and S1B). A total of 31 genes were enriched in these two pathways. The 31 genes are positively regulated by TGFβ1 and are involved in signaling and cellular interactions in the TME. Overlap of the genes associated with prognosis showed that LAMC1 was the only one of the 31 genes that affected the prognosis of ESCC patients (Fig. 1E). Accordingly, at the protein level, LAMC1 was more highly expressed in cancer tissues than in para‐cancer tissues, as demonstrated by IHC staining (Fig. 1F) and was also associated with low OS (Fig. 1G) and tumor stage (Table S1). Therefore, LAMC1 can be used as an independent prognostic marker for ESCC.

Compared with the control group, LAMC1 expression was increased at the protein and mRNA levels in KYSE30 and KYSE450 cells after TGFβ1 treatment, which could be time‐ and concentration‐dependent in ESCC cells (Figs 1H and S2A,B). Additionally, to determine whether the TGFβ signaling pathway is responsible for LAMC1 expression, we used the TGFβ receptor inhibitor SB505124 to eliminate the effect of TGFβ1 on LAMC1. The results showed that SB505124 could reverse TGFβ1‐induced LAMC1 expression in KYSE30 and KYSE450 cells (Fig. 1I,J), suggesting that TGFβ signaling is responsible for the induction of LAMC1 transcription. From the database of Human Transcription Factor Targets (hTFtarget) prediction [36], we speculated that the transcription factors SMAD4 and SP1 synergistically induced LAMC1 transcription (Tables S2 and S3, Fig. S3). First, we detected SMAD4 and SP1 protein levels under TGFβ1 treatment, and found that the expression of SMAD4 and SP1 could be upregulated after TGFβ1 in wild‐type KYSE30 and KYSE450 cells, which could be reversed by the TGFβ receptor inhibitor SB505124 (Fig. S4). A ChIP assay was then performed using anti‐SMAD4 and anti‐SP1 antibodies and we found that TGFβ1 led to a significant increase in the enriched LAMC1 promoter sequence, suggesting that SMAD4 and SP1 were recruited to the promoter of the LAMC1 gene by TGFβ1 treatment (Fig. 1K). Additionally, by measuring proteins of chromatin fractions with antibodies against SP1 and SMAD4, we found that the expression of SMAD4 and SP1 was increased in the other’s chromatin fraction (Fig. 1L). We also knocked down SP1 or SMAD4 in ESCC cells, and established ESCC cells with the combined knockdown (Figs 1M,N and S5A,B). LAMC1 expression was decreased in these cells and could not be rescued by TGFβ1 treatment (Fig. 1O–Q). This suggests that the transcription factors SP1 and SMAD4 together induced the transcription of LAMC1. The above results showed that LAMC1 was directly regulated by the TGFβ/SMAD4‐SP1 signaling pathway.

### 3.2 LAMC1 promotes the proliferation and migration of ESCC cells *in vitro* and *in vivo*

**Fig. 2:**
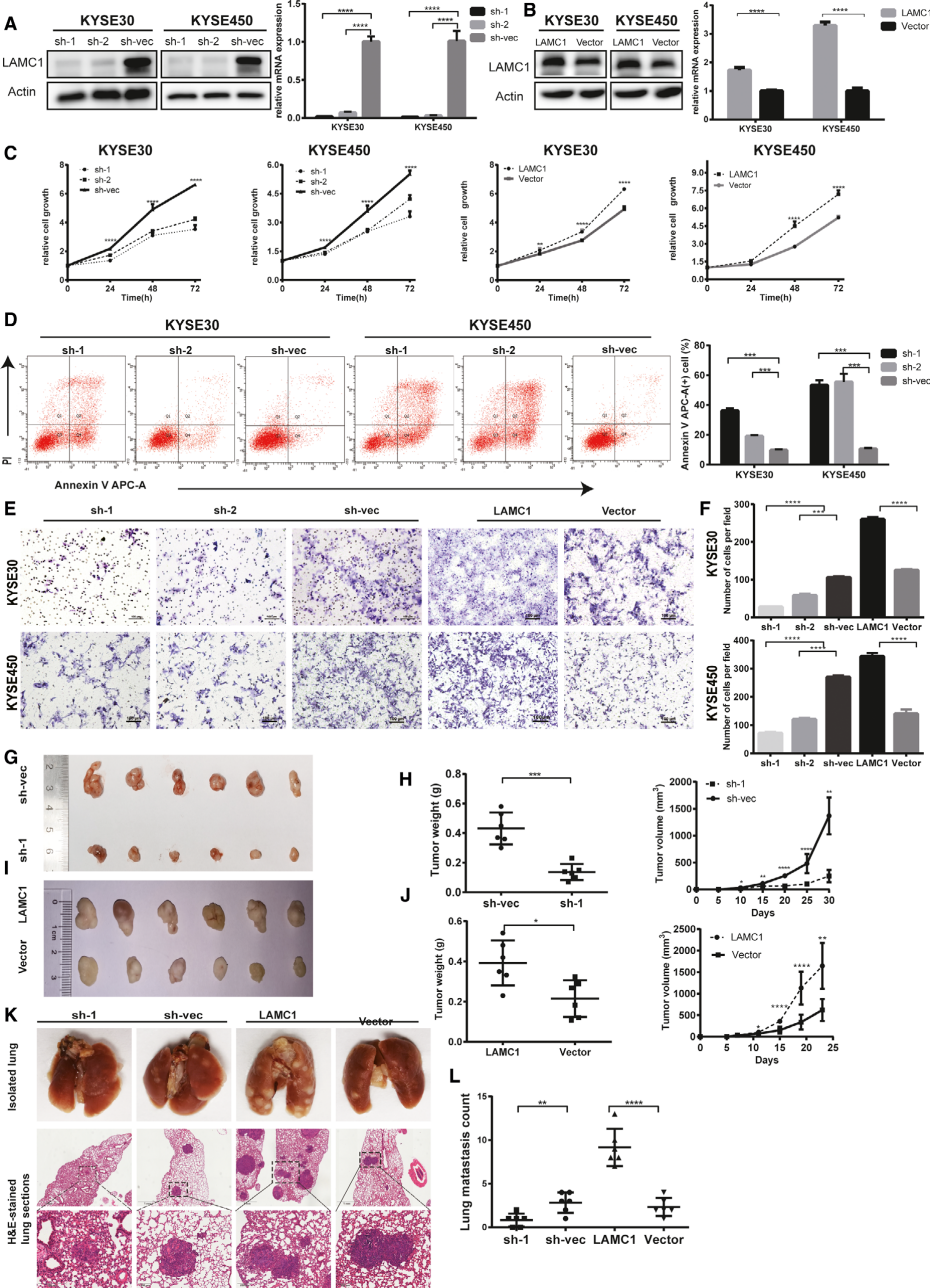
LAMC1 promoted the proliferation and metastasis of ESCC cells *in vivo* and *in vitro*. (A,B) Knockdown or overexpression efficiency of LAMC1 in ESCC cells was verified by western blot and RT‐qPCR. (C) The rate of cell growth of KYSE30 and KYSE450 cells treated with shLAMC1 or overexpressing LAMC1 and vector control cells was measured by CCK‐8 assay. (D) Apoptosis of shLAMC1‐1, ‐2 and sh‐vec KYSE30 and KYSE450 cells was analyzed using the Annexin V APC Apoptosis Detection Kit. (E,F) The migration ability of shLAMC1‐1, ‐2, sh‐vec, LAMC1 and vector KYSE30 and KYSE450 cells was detected by chamber assay. The numbers of migrating cells were compared between the groups. (G–J) Representative images of tumor formation in nude mice subcutaneously inoculated with KYSE30 cells stably expressing LAMC1 shRNA or negative control and expressing LAMC1 or mock‐vehicle control (G, I), and tumor weights and volumes for groups (H, J). (K,L) Representative images of lung tissues isolated from mice injected with 1 × 10^6^ shLAMC1, sh‐vec, LAMC1 or vector KYSE30 cells via the tail vein and hematoxylin and eosin stained images (200×) of such tissues, and quantification of lung metastasis (scale bars: 1 mm and 200 µm for the top and bottom rows of photomicrographs, respectively). Three biological replicates were performed for *in vitro* assays. The data in bar charts are presented as the mean ± SD. *P < 0.05, **P < 0.01, ***P < 0.001, ****P < 0.0001 (Student’s *t*‐test).

To evaluate the tumorigenic effect of LAMC1 on ESCC, we constructed KYSE30 and KYSE450 cell lines with stable knockdown (Fig. 2A) or overexpression of LAMC1 (Fig. 2B). The shLAMC1 in KYSE30 and KYSE450 cells inhibited cell proliferation. Accordingly, overexpression of LAMC1 promoted cell proliferation (Fig. 2C). The shLAMC1 in KYSE30 and KYSE450 cells promoted apoptosis (Fig. 2D). Furthermore, the overexpression of LAMC1 promoted ESCC cell migration, whereas shLAMC1 inhibited migration (Fig. 2E).

*In vivo*, we established a xenograft tumor mouse model by subcutaneous inoculation or intravenous tail injection of KYSE30 cells transfected with shLAMC1, sh‐vec, overexpressed LAMC1 and control vector. Consistent with the results of the *in vitro* experiments, the tumor volume and weight in the LAMC1‐overexpression group were significantly increased compared with those in the control group. The shLAMC1 group exhibited the opposite pattern (Fig. 2G–J). The number of pulmonary metastasis nodules in the groups was similar (Fig. 2K,L).

### 3.3 The positive effect of LAMC1 on the migration of ESCC cells mainly occurs via the Akt/IKKα/NF‐κB/MMP9‐MMP14 pathway

**Fig. 3:**
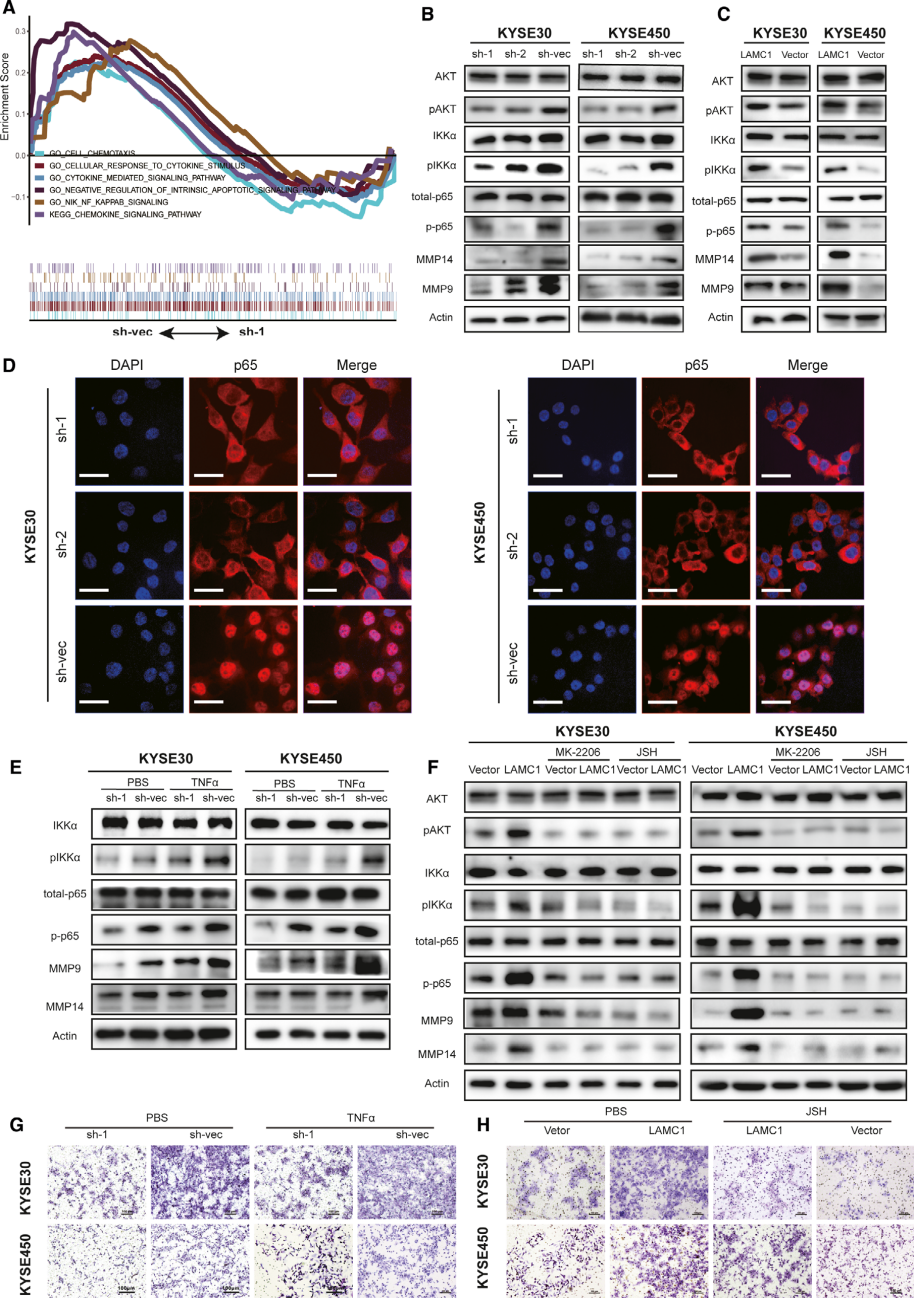
The positive effect of LAMC1 on ESCC cell migration occurred mainly via the Akt/IKKα/NF‐Κb/MMP9/14 pathway. (A) Antiapoptosis, NF‐κB and cytokine‐related pathways enriched by sh‐vec using GSEA. (B,C) Expression levels of phosphorylated Akt, NF‐κB, IKKα, MMP9 and MMP14 in KYSE30 and KYSE450 cells stably expressing LAMC1 shRNA or negative control and expressing LAMC1 or mock‐vehicle control. (D) Immunofluorescence staining of p65 in shLAMC1‐1, shLAMC1‐2, sh‐vec KYSE30 and KYSE450 cells. Nuclei were visualized with DAPI staining (blue). Representative images are shown. Scale bar: 34 μm. (E,F) Phosphorylation of IKKα, p65, MMP9 and MMP14 was also detected in shLAMC1 KYSE30 and KYSE450 cells treated with or without TNFα stimulation (E), and overexpressing LAMC1 with or without 5 μm Akt phosphorylation selective inhibitor MK‐2206 2HCI and 10 μm NF‐κB JSH‐23 stimulation (F). (G,H) Representative images of migration of these subgroups, shLAMC1 with or without TNFα (10 ng·mL^−1^) stimulation, overexpression of LAMC1 with or without JSH‐23 stimulation, measured by chamber assay. Three biological replicates were performed for *in vitro* assays.

We performed mRNA sequencing in KYSE30 and KYSE450 cells with sh‐1 LAMC1 or sh‐vec to explore downstream signaling pathways responsible for the aggressiveness of ESCC. GSEA suggested that LAMC1 knockdown could affect the apoptosis pathway, the NF‐κB pathway, and cytokine and chemokine pathways (Fig. 3A). We detected IKKα phosphorylation of Akt, IKKα and p65 levels in shLAMC1‐ and LAMC1‐overexpressing ESCC cells and found that LAMC1 expression was positively correlated with the phosphorylation of Akt, IKKα and p65 (Fig. 3B,C). Matrix metalloproteinases (MMP) play an important role in tumor cell invasion and metastasis, and are common downstream regulators of NF‐κB‐mediated cell metastasis [37]. The expression of MMP9 and MMP14 was in accordance with the phosphorylation of Akt, IKKα and p65 in shLAMC1‐expressing and LAMC1‐overexpressing ESCC cells (Fig. 3B,C). Furthermore, through cell immunofluorescence, we found that the expression of NF‐κB (p65) was decreased in the nucleus of LAMC1 knockdown cells compared with the controls (Fig. 3D). Additionally, TNFα, as an activator of the NF‐κB pathway, could restore the phosphorylation of IKKα, NF‐κB, MMP9 and MMP14 in LAMC1 knockdown cells (Fig. 3E). Accordingly, the Akt phosphorylation selective inhibitorMK‐2206 and the NF‐κB nuclear translocation inhibitor JSH‐23 both reversed the high expression of phosphorylated Akt, IKKα, NF‐κB, MMP9 and MMP14 in LAMC1‐overexpressing cells (Fig. 3F). TNFα reversed the inhibitory migration of shLAMC1 ESCC cells as well (Figs 3G and S6A). Accordingly, JSH‐23 abrogated the promotion of migration in LAMC1‐overexpressing ESCC cells (Figs 3H and S6B).

### 3.4 LAMC1 inhibits apoptosis mainly through the Akt/NF‐κB/caspase9‐caspase3‐PARP cascade

**Fig. 4:**
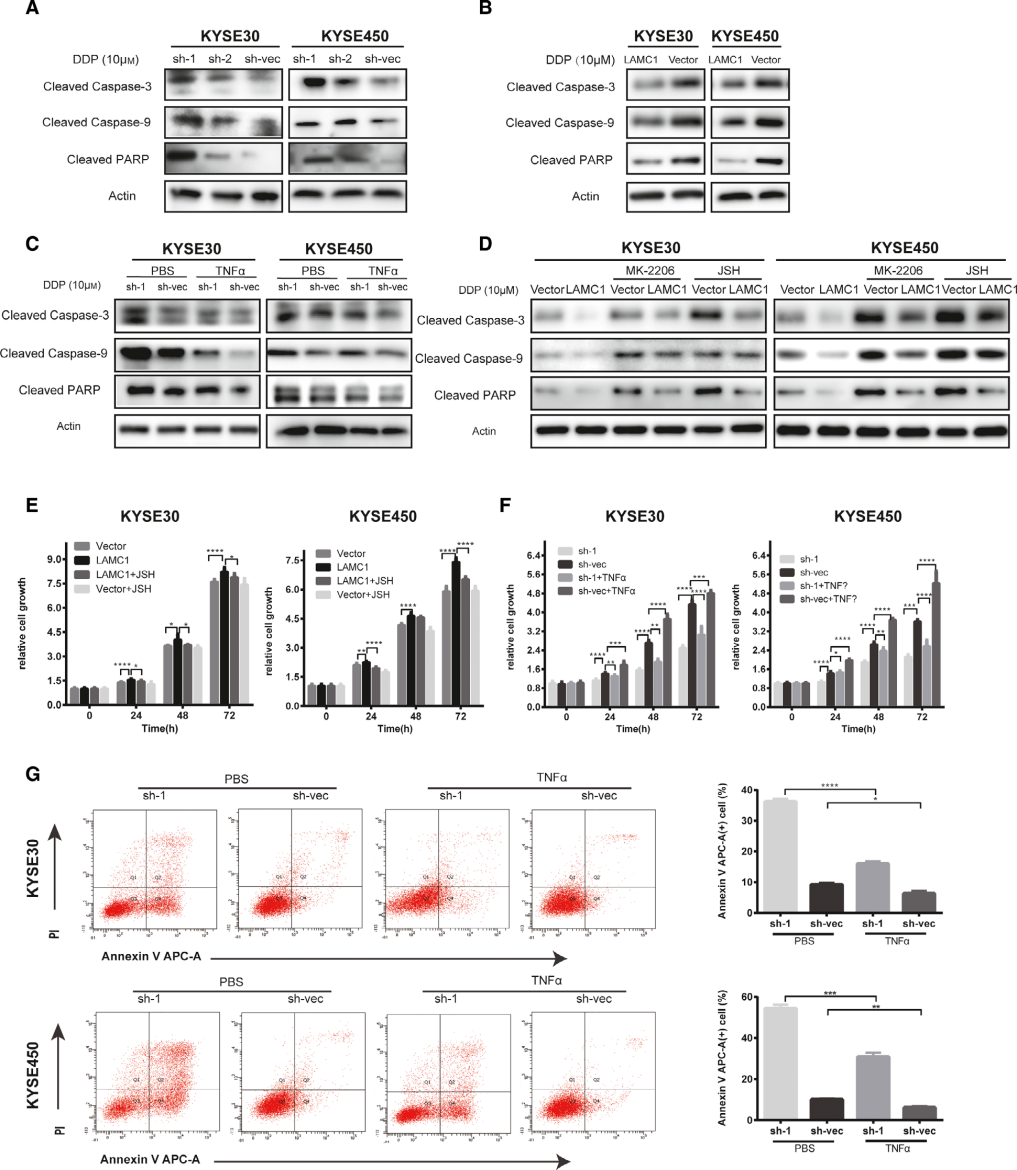
LAMC1 inhibited apoptosis possibly through the Akt/NF‐κB/caspase9‐caspase3‐PARP cascade. (A,B) Western blotting was conducted to detect the protein levels of cleaved caspase‐3, caspase‐9, and PARP in KYSE30 and KYSE450 cells stably expressing LAMC1 shRNA or negative control and expressing LAMC1 or mock‐vehicle control, which were induced by 10 µm cisplatin for 24 h. (C,D) After cisplatin induction (10 mm for 24 h), cleaved caspase‐3, caspase‐9 and PARP were detected in KYSE30 and KYSE450 cells treated with shLAMC1 with or without TNFα stimulation (C) or overexpressing LAMC1 with or without 5 μm Akt phosphorylation selective inhibitor MK‐2206 2HCI and 10 μm NF‐κB nuclear translocation inhibitor JSH‐23 stimulation (D). (E–G) Proliferation and apoptosis of KYSE30 and KYSE450 cells stably expressing LAMC1 shRNA or negative control and expressing LAMC1 or mock‐vehicle control with or without TNFα or JSH‐38 (JSH), as measured by a CCK8 assay. Three biological replicates were performed for *in vitro* assays. The data in bar charts are presented as the mean ± SD. *P < 0.05, **P < 0.01, ***P < 0.001, ****P < 0.0001 (Student’s *t*‐test).

Cleaved caspase‐9, cleaved caspase‐3 and cleaved PARP levels were increased in shLAMC1 KYSE30 and KYSE450 cells compared with the control group after cisplatin treatment (Fig. 4A) and were decreased in LAMC1‐overexpressing cells compared with vector‐only cells (Fig. 4B). Furthermore, previous studies have shown that the NF‐κB pathway can regulate cell anti‐apoptosis via caspase [38]. We found that the expression of cleaved caspase‐9, cleaved caspase‐3, and cleaved PARP in shLAMC1 cells was decreased by TNFα stimulation (Fig. 4C). Accordingly, after treatment with the Akt phosphorylation selective inhibitor MK‐2206 and the NF‐κB nuclear translocation inhibitor JSH‐23, LAMC1‐overexpressing ESCC cells showed the opposite results in proliferation and expression of cleaved caspase and PARP (Fig. 4D). Furthermore, JSH‐23 also restored the positive effect on the proliferation of LAMC1‐overexpressing cells (Fig. 4E), and TNFα restored the negative effect on proliferation and the positive effect on the apoptosis of shLAMC1 KYSE30 and KYSE450 cells (Fig. 4F,G).

### 3.5 CXCL1 is regulated by LAMC1 mainly via NF‐κB activation

**Fig. 5:**
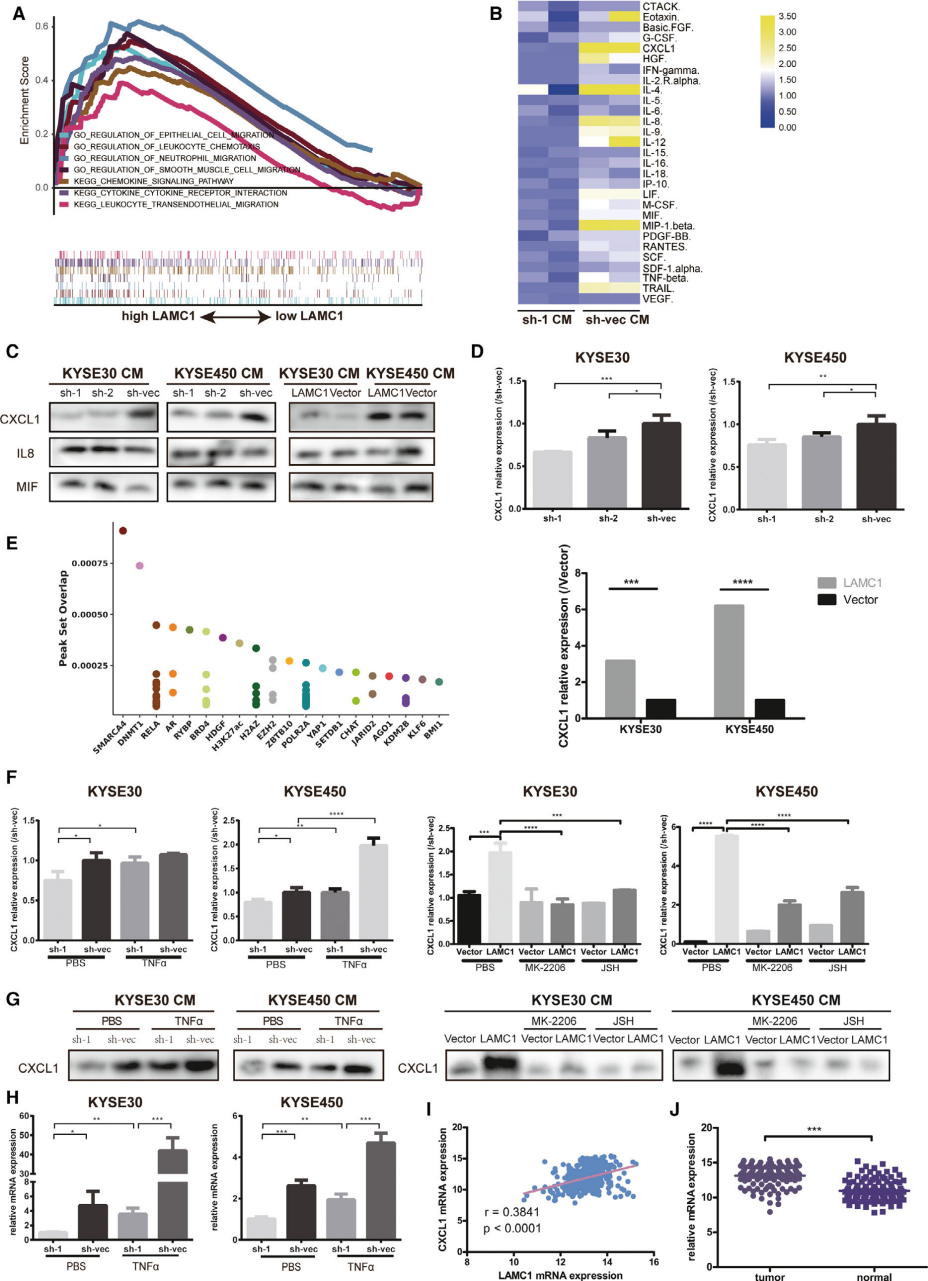
LAMC1 promoted CXCL1 secretion mainly by activating NF‐κB. (A) LAMC1 affected the cytokine and chemokine signaling pathways according to GSE53625 data. (B) A 48‐cytokine panel was used to detect cytokines in the CM of shLAMC1 and sh‐vec KYSE450 cells. (C) Expression of three cytokines in the CM of KYSE30 and KYSE450 cells stably expressing LAMC1 shRNA or negative control and expressing LAMC1 or mock‐vehicle control by western blot. (D) CXCL1 expression in the CM of shLAMC1 and LAMC1‐overexpressing KYSE30 and KYSE450 cells, as measured by ELISA. (E) Top 20 predicted transcription factors of CXCL1 in the Cistome network. (F,G) ELISA (F) and western blotting (G) were conducted to detect CXCL1 expression in the CM of shLAMC1 and sh‐vec cells with or without TNFα treatment, and of LAMC1‐overexpressing cells with or without 5 μm Akt phosphorylation selective inhibitor MK‐2206 2HCI and 10 μm NF‐κB nuclear translocation inhibitor JSH‐23 stimulation. (H) RT‐qPCR was conducted to detect CXCL1 expression in shLAMC1 and sh‐vec cells with or without TNFα treatment. (I) LAMC1 was positively associated with CXCL1 based on GSE53625 data. (J) CXCL1 was upregulated in tumor tissue compared with that in adjunct tissue in the GSE53625 dataset. Three biological replicates were performed for *in vitro* assays. The data in bar charts are presented as the mean ± SD. *P < 0.05, **P < 0.01, ***P < 0.001, ****P < 0.0001 (Student’s *t*‐test).

Increasing attention has been given to the role of the TME in solid tumors. Cytokines and chemokines, as tumor promoting factors, often play a role in intercellular signaling. By GSEA enrichment analysis of mRNA‐seq data of shLAMC1 and sh‐vec ESCC cells, knockdown of LAMC1 affected cytokine and chemokine signaling pathways (Fig. 3A). Moreover, enrichment analysis of RNA‐seq data of ESCC tissues (GSE53625) revealed the same effect as LAMC1 (Fig. 5A). We hypothesized that LAMC1 is involved in the regulation of signaling molecules and interactions in the TME via enrichment analysis of genes positively regulated by TGFβ1. All the above findings suggest that LAMC1 may be involved in regulating the secretion of cytokines or chemokines. First, we detected a total of 48 cytokines and chemokines in the CM of shLAMC1 cells using the Bio‐Plex Pro Human Chemokine Panel 48‐plex kit. We found that the expression of CXCL1, IL8 and macrophage migration inhibitory factor (MIF) was increased in sh‐vec cells compared with in sh‐1 cells at a higher concentration (Fig. 5B). We also detected the expression of the three cytokines in concentrated CM and found that only CXCL1 was regulated by LAMC1 in both KYSE30 and KYSE450 cells (Fig. 5C). Considering these results, we speculate that LAMC1 may upregulate CXCL1, which was confirmed in LAMC1‐knockdown and LAMC1‐overexpression cells by ELISA (Fig. 5D). Thus, CXCL1 could be identified as a downstream target of LAMC1.

As predicted on the Cistrome website [39], NF‐κB can upregulate CXCL1 through transcriptional activation (Fig. 5E). In addition, we determined that LAMC1 may activate the NF‐κB pathway; thus, we speculated that LAMC1 upregulates CXCL1 through NF‐κB transcriptional activation. To verify whether NF‐κB is responsible for CXCL1 expression, we used ELISA and WB to detect CXCL1 secretion in LAMC1‐knockdown cells with or without TNFα stimulation and in LAMC1‐overexpression cells with or without MK‐2206 2HCI and JSH‐23. As expected, reduced CXCL1 secretion by LAMC1‐knockdown cells was increased by TNFα, and MK‐2206 2HCI and JSH‐23 reversed the high CXCL1 expression of LAMC1‐overexpression cells (Fig. 5F,G). At the mRNA level, the expression of CXCL1 in shLAMC1 cells was also reversed by TNFα (Fig. 5H).

*In vivo*, LAMC1 expression was associated with that of CXCL1 at the RNA level (Fig. 5I). CXCL1 expression was higher in cancer tissues than in adjacent tissues (Fig. 5J) but did not affect patient prognosis, specifically OS (Fig. S7E).

### 3.6 CXCL1 secreted by ESCC tumor cells promotes the transformation of CAF into inflammatory CAF

**Fig. 6:**
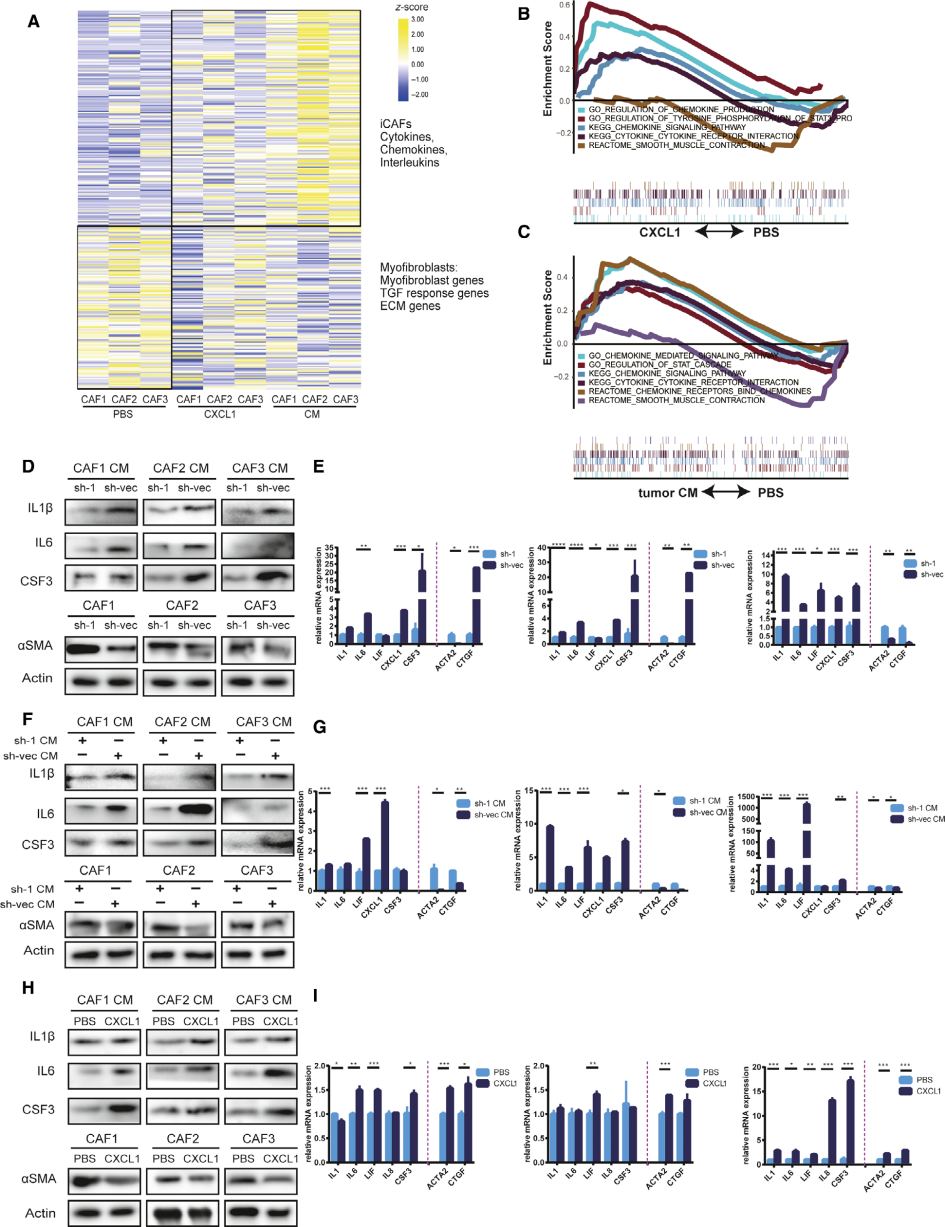
Tumor‐secreted CXCL1 promoted the formation of inflammatory CAF (iCAF). (A) Transcriptional profile of CAF with or without treatment with recombinant CXCL1 (rCXCL1) and sh‐vec conditioned medium (CM). (B,C) rCXCL1 or sh‐vec CM promoted activation of cytokine and chemokine pathways and phosphorylation of STAT3, as demonstrated by GSEA. (D–I) Western blot and RT‐qPCR detection of markers of iCAF and myofibroblasts (myCAF), respectively, in CM of CAF cocultured with shLAMC1 or sh‐vec ESCC cells (D,E), treated with the CM of shLAMC1 or sh‐vec ESCC cells (F,G), or treated with rCXCL1 (H,I). Three biological replicates were performed for *in vitro* assays. The data in bar charts are presented as the mean ± SD. *P < 0.05, **P < 0.01, ***P < 0.001, ****P < 0.0001 (Student’s *t*‐test).

CAF are heterogeneous cells with different subtypes, such as iCAF and myCAF [17]. The two subgroups not only have significant differences in their transcriptional profiles but also have different effects on tumor cells. myCAF are contractile and can remodel the stroma, and iCAF are characterized by a secretory phenotype and regulate tumor cells and other cells in a paracrine manner. CAF were isolated from fresh tumor tissue and cultured *in vitro* and were identified by αSMA expression by cell immunofluorescence detection (Fig. S8A). We detected the expression of the markers of iCAF or myCAF in CAF derived from ESCC samples. The results showed that CAF derived from ESCC samples had different expression levels of those markers (Fig. S8B), which suggested that CAF had heterogeneity in ESCC. To explore whether ESCC tumor cell‐secreted CXCL1, upregulated by LAMC1, influences CAF heterogeneity, we performed mRNA‐seq in the following cells: CAF treated with PBS; CAF with recombinant CXCL1 (rCXCL1) treatment; CAF with sh‐vec CM treatment. We found that in CAF treated with rCXCL1 or sh‐vec CM, some gene clusters of iCAF, including cytokines (CSF2, VEGF, etc. ), chemokines (CXCL2, CXCL3, CXCL5, etc.) and interleukins (IL6, IL7, etc.), were upregulated compared with CAF treated with PBS. In addition, gene clusters of myCAF, such as COL1A1 and COL4A1, were downregulated (Fig. 6A). Moreover, compared with analysis of the controls, GSEA of CAF with rCXCL1 or sh‐vec CM treatment confirmed the upregulation of cytokine/chemokine signaling and the regulation of the STAT cascade, especially phosphorylation of the STAT3 pathway. However, the smooth muscle contraction pathway was downregulated (Fig. 6B,C).

To verify the above sequencing results, we detected iCAF markers (IL1, IL6, LIF, CSF3) and myCAF markers (Acta2, Ctgf) in CAF treated with CM from shLAMC1 ESCC cells, CAF cocultured with shLAMC1 ESCC cells, and controls. Because inflammatory markers are secreted, the expression of these proteins was detected in the concentrated CM of CAF. At the protein and mRNA levels, we found that CAF cocultured with sh‐vec ESCC cells or treated with the CM of sh‐vec ESCC cells had higher expression of inflammatory markers compared with CAF cocultured with shLAMC1 ESCC cells (Fig. 6D,E) or treated with CM from shLAMC1‐1 ESCC cells (Fig. 6F,G). However, myCAF markers (Acta2 and Ctgf) had lower expression in CAF cocultured with sh‐1 ESCC cells or treated with the CM of sh‐1 ESCC cells compared with controls at the protein level, which was not obvious at the mRNA level (Fig. 6G). To verify whether CXCL1 is the main driver of this effect, we directly treated CAF with rCXCL1 and obtained a similar result (Fig. 6H,I).

### 3.7 Tumor‐secreted CXCL1 induces iCAF formation via the phosphorylation of STAT3

**Fig. 7:**
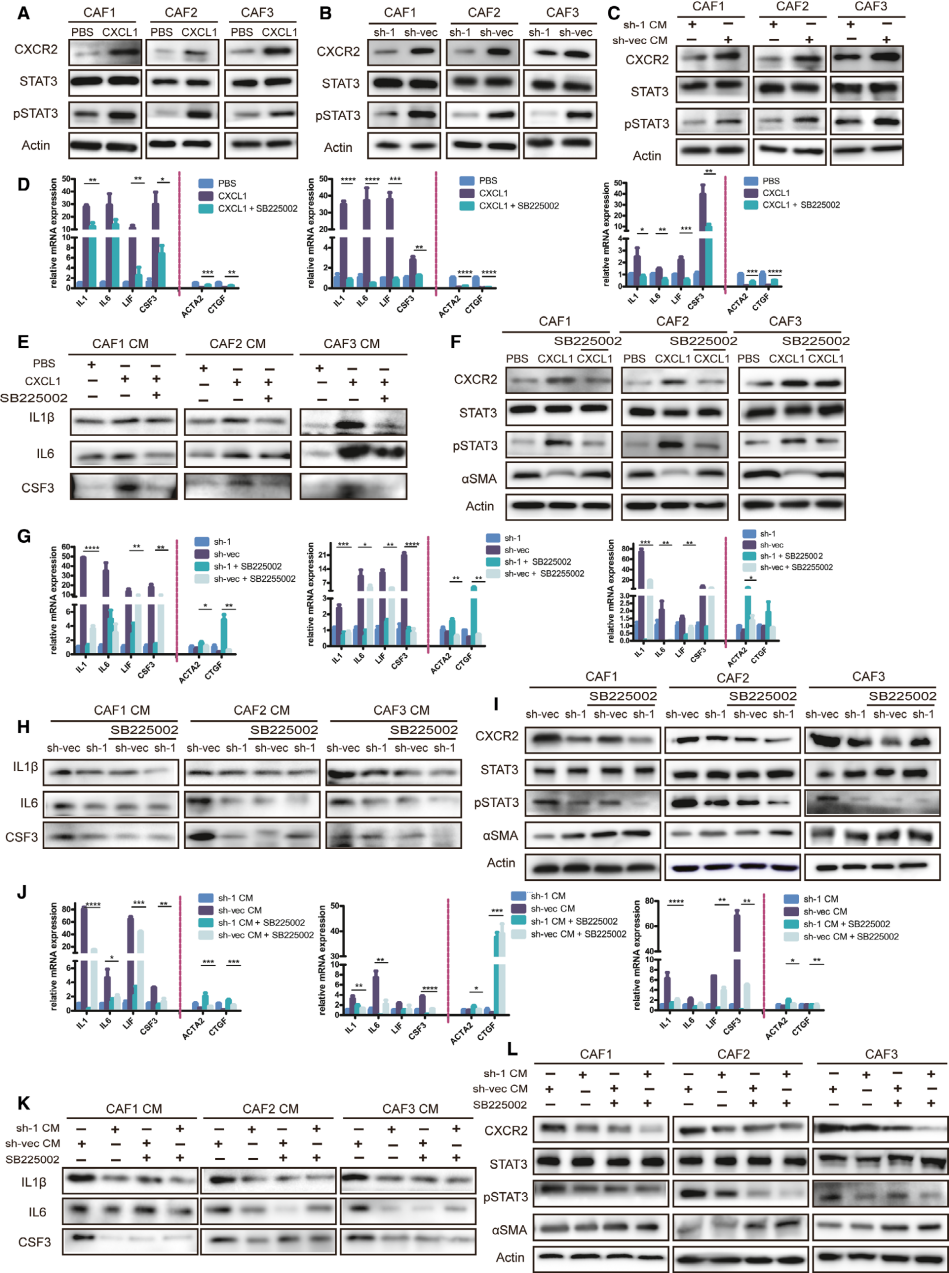
CXCL1 induced inflammatory CAF formation via phosphorylation of STAT3. (A‐C) Expression of pSTAT3 and CXCR2 was detected in CAF with three different treatments: induced with rCXCL1 (A), cocultured with shLAMC1 and sh‐vec KYSE30 cells (B) or stimulated by CM from these tumor cells (C), as demonstrated by western blot. (D–I) IL1β, IL6 and CSF3 were detected by western blot and RT‐qPCR in concentrated CM of CAF with three treatments with or without the CXCR2 inhibitor SB225002 (D,E,G,H,J,K), and αSMA, pSTAT3 and CXCR2 were detected in the total protein of cells (F,I,L). Three biological replicates were performed for *in vitro* assays. The data in bar charts are presented as the mean ± SD. *P < 0.05, **P < 0.01, ***P < 0.001, ****P < 0.0001 (Student’s *t*‐test).

The CXCL1 common receptor is CXCR2; combined with the above GSEA results, we suggest that the CXCR2/pSTAT3 pathway may be responsible for this effect. We found that CXCR2 and pSTAT3 expression in CAF was upregulated at the RNA and protein levels after rCXCL1 treatment (Figs 7A and S9). Additionally, SB225002, an inhibitor of CXCR2, reversed the upregulation of the inflammatory markers, CXCR2 and pSTAT3 and the downregulation of αSMA by rCXCL1 in CAF (Fig. 7D–F). In addition, SB225002 decreased the changes in these proteins, especially inflammatory markers, in CAF cocultured with shLAMC1 ESCC cells and CAF treated with CM from shLAMC1 ESCC cells (Fig. 7B–C, G–L). The above results suggest that CXCL1, regulated by LAMC1 and secreted by tumor cells, promotes the transition of CAF into iCAF via CXCR2/pSTAT3.

### 3.8 iCAF induced by CXCL1 promotes ESCC progression *in vivo* and *in vitro*

**Fig. 8:**
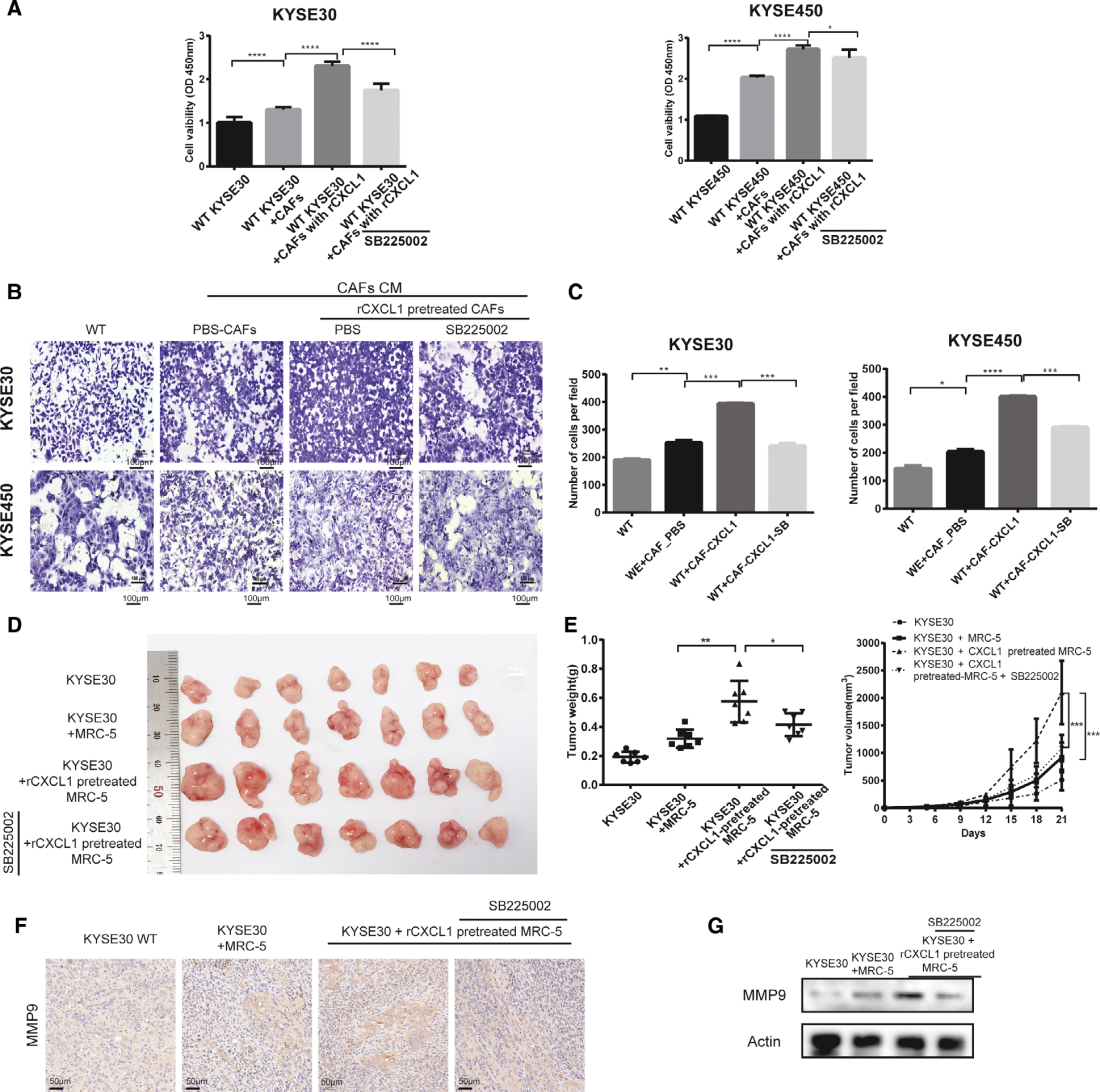
Inflammatory CAF, induced by CXCL1, promoted the proliferation and migration of ESCC *in vitro* and *in vivo*. (A) Detection of the viability of WT KYSE30 and KYSE450 cells cocultured for 48 h with or without PBS‐treated CAF, CXCL1‐induced CAF, or with SB225002, as demonstrated by CCK‐8 assay. (B,C) Migration of WT KYSE30 cells with different treatments: with or without CM of CAF that were or were not pre‐induced with rCXCL1 combined with SB225002, as measured by chamber assay. (D,E) Comparison of tumor volume and weights of subcutaneous tumor xenografts established from the co‐implantation of KYSE30 cells and CXCL1‐pretreated or not pretreated MRC‐5 cells in the presence or absence of SB225002 treatment. (F) Representative immunohistochemical (IHC) images of MMP9 staining in xenograft tumor tissue of WT KYSE30 cells admixed with implanted CAF with different pretreatments (original magnification: 200x). (G) Expression of MMP9 in xenograft tumor tissue of WT KYSE30 cells admixed with implanted CAF with different pretreatments was detected by western blot. Three biological replicates were performed for *in vitro* assays. The data in bar charts are presented as the mean ± SD. *P < 0.05, **P < 0.01, ***P < 0.001, ****P < 0.0001 (Student’s *t*‐test).

To explore the effect of iCAF induced by CXCL1 on the proliferation of tumor cells, we compared tumor cell proliferation after coculture with CAF stimulated by PBS or rCXCL1 together with or without SB225002. We found that CAF with rCXCL1 promoted WT ESCC proliferation, which could be reversed by SB225002 (Fig. 8A). Additionally, to explore the influence of iCAF on migration, WT ESCC cells with different treatments were divided into four groups: the control group (WT ESCC cells treated with PBS) and WT ESCC cells treated with CM secreted by CAF that were pretreated with rCXCL1 or PBS (CM‐CAF‐PBS, CM‐CAF‐pretreatCXCL1) in the presence or absence of SB225002. We found that both CM‐CAF‐pretreated CXCL1 and CM‐CAF‐PBS could promote the proliferation and migration of ESCC cells, and CM‐CAF‐pretreated CXCL1 had a stronger promoting effect than CM‐CAF‐PBS, whereas SB225002 could reverse the effect (Fig. 8B,C). *In vivo*, the tumor volume and weight of WT KYSE30 cells mixed with rCXCL1‐pretreated CAF were larger than those of cells mixed with CAF without rCXCL1 pretreatment; SB225002 weakened this effect (Fig. 8D,E). The migration marker MMP9 was measured by IHC and western blot in xenograft tumor tissue. Tumors pretreated with rCXCL1 had higher MMP9 expression than did tumors pretreated with PBS, and MMP9 expression was decreased by SB225002 (Fig. 8F,G).

## 4 Discussion

As in other types of tumors, high LAMC1 expression can promote ESCC tumor cell proliferation and migration and is associated with a poor prognosis; thus, LAMC1 can be used as a biomarker. We found that TGFβ1 regulates LAMC1 expression via SMAD4/SP1 synergistic activation. Previous studies have shown that LAMC1 is regulated by SP1 in hepatocellular carcinoma [40, 41, 42]. The mechanism by which SMAD and SP1 synergistically activate genes has been reported previously [43].

The positive effect of LAMC1 on Akt phosphorylation‐mediated NF‐κB activation in ESCC could promote the downstream antiapoptotic, promigratory and secretion processes of CXCL1. Many previous studies also confirmed the high expression of NF‐κB in ESCC [34]. NF‐κB expression was increased in mouse models of ESCC with p120‐catenin knockdown. NF‐κB signals are activated by regulating upstream mediators, such as upregulating the transcription factor Id‐1 or downregulating the tumor suppressor Nkx2‐8 [3]. TGFβ‐induced long noncoding RNAs repress NF‐κB signaling [34]. We found that shLAMC1 ESCC cells had higher expression of cleaved caspase 9, caspase 3 and PARP. The apoptosis pathways are mainly divided into endogenous and exogenous pathways, and the activation of caspase 9 and caspase 8, which are apoptotic initiators, represents endogenous and exogenous apoptosis initiation, respectively. After cleavage and activation, caspase enzymes, such as caspase 3, are activated in a cascade manner, and cytoskeletal and nuclear proteins, such as PARP, are cleaved to promote apoptosis. LAMC1 could promote ESCC migration mainly by upregulating MMP9 and MMP14 downstream of NF‐κB. In ESCC, certain MMPs are upregulated to promote invasion, such as MMP2, MMP7 and MMP9 [37, 44]. In addition, LAMC1 and MMP are both components of the ECM and are related to ECM remodeling. ECM remodeling plays an important role in the development of tumors, especially in invasion [45]. Other components of the ECM have also been reported in ESCC, such as fibronectin, proteoglycan dermatan sulfate and hyaluronan [46]. LAMC1 can promote CXCL1 secretion through the transcriptional activation of NF‐κB and, as a transcription factor, NF‐κB can regulate cytokine production [47]. Activation of the NF‐KB pathway is often considered an important link between the inflammatory microenvironment and tumor development [47]. The cytokines downstream of NF‐κB in ESCC have been reported to be IL8 and IL1 [48, 49, 50]. IL1 promotes tumor invasion, tumor‐mediated immunosuppression and tumor stem cell self‐renewal [51]. IL1 also plays a role in inducing iCAF [19].

Many previous studies of LAMC1 have indicated that high LAMC1 expression can serve as a biomarker for a variety of tumors [28], but less attention has been given to the effect of LAMC1 on the heterogeneity of CAF. We determined that ESCC tumor cells secrete CXCL1 and promote iCAF activation. In addition, we verified that TGFβ1 upregulated LAMC1. Furthermore, we found that CXCL1 expression was increased with TGFβ1 treatment, which could be eliminated by the TGFβR1 inhibitor SB505124 (Fig. S7A–C). TGFβ1 was positively associated with CXCL1 based on GSE53625 data (Fig. S7D). Consistent with our findings, in previous studies, CXCL1 expression increased in cocultures of CAF and oral squamous cell carcinoma cells with IL1β, promoting tumor invasion and CAF activity [52]. In addition, CAF‐secreted CXCL1 can induce ESCC progression [53]. It is worth mentioning that CXCR2 inhibitors showed a better response to rCXCL1 on CAF than tumor CM, suggesting that LAMC1 may also influence other factors contributing to the formation of iCAF. Whether LAMC1 influences the heterogeneity of CAF by affecting other factors or exosome secretion in tumor cells may require further study. Compared with sh‐vector, the changes of IL8 and MIF in shLAMC1 CM were obvious in the 48 cytokines detected; we also detect the effects of IL8 and MIF on the heterogeneity of CAF, but there was no consistent conclusion (Fig. S10). Furthermore, iCAF and myCAF are converted into each other [19]. We found that regardless of the type of CAF derived from ESCC samples, CXCL1 increased the expression of inflammatory markers in CAF and decreased the expression of myofibroblast markers. In accordance with previous studies, iCAF stimulated by rCXCL1 and sh‐vec CM also presented a secretory phenotype that can interact with other cells in a paracrine manner and have tumor‐promoting functions [17]. Due to its secretory phenotype, we speculate that iCAF induced by CXCL1 may be involved in cancer‐associated systemic effects [17]. CXCL1 activates the phosphorylation of STAT3 in CAF, which leads to an increase in many inflammatory mediators. Similarly, in pancreatic ductal cell carcinoma, tumor cell‐secreted IL1 induces the formation of iCAF in a cascade involving increased LIF expression and activation of JAK/STAT signaling [19]. It has also been confirmed that CAF can reprogram cytokine secretion by other cells [23]. Moreover, it is worth noting that CAF stimulation by either rCXCL1 or tumor CM did not induce proliferation (Fig. S11). This further indicated that the carcinogenic effect of CAF was not through its own proliferation but through the indirect regulation of the phenotype of tumor cells through paracrine signaling [6, 10].

We confirmed that TGFβ1 acted on tumor cells, causing a series of changes: upregulation of LAMC1; phosphorylation of NF‐κB; secretion of CXCL1; phosphorylation of STAT3 in CAF; and finally, the induction of the formation of iCAF, that is, tumor‐promoting CAF. Previous studies have shown that TGFβ signaling plays a dual role in CAF [26]. It can activate CAF, e.g. through the conversion of NF into CAF and fibroblasts into tumor‐promoting CAF [23, 26]. However, TGFβ1 secreted by tumor cells inhibits iCAF formation by antagonizing IL1 signaling activity and prevents myCAF conversion to iCAF by blocking the JAK/STAT pathway [19]. These dual functions may be due to the balance between TGFβ signaling and STAT signaling in CAF [19, 54, 55].

## 5 Conclusion

In conclusion, the overexpression of LAMC1, upregulated by TGFβ1 via SMAD4/SP1 synergistic activation, promoted the proliferation and migration of tumor cells mainly via NF‐κB/MMP9‐MMP14. Additionally, LAMC1 facilitated CXCL1 secretion via NF‐κB. Tumor‐secreted CXCL1 remodeled the formation of iCAF through CXCR2/pSTAT3. The CM of iCAF promoted the proliferation and migration of tumor cells.

## Conflict of interest

The authors declare no conflict of interest.

## Data accessibility

The data that support the findings of this study are available from the corresponding author (prof.jiehe@gmail.com) upon reasonable request.

## Authors’ contributions

LLF, NS and JH conceived the ideas and designed the experiments. LLF, YC, CQZ, JBH, YYL and ZLL performed the experiments. YC and NS provided the clinical samples. LLF analyzed study data. LLF wrote the paper. JH reviewed the manuscript. All authors read and approved the final manuscript.

## Ethics approval and consent to participate

All patients from whom CAF were isolated, signed informed consent forms. Our study was approved by the Committee for the Ethics Review of Research Involving Human Subjects of the Cancer Hospital of the Chinese Academy of Medical Sciences. All animal experiments were approved by the Institutional Animal Care and Use Committee of the Cancer Hospital, Chinese Academy of Medical Sciences and Peking Union Medical College.

### Peer Review

The peer review history for this article is available at https://publons.com/publon/10.1002/1878‐0261.13053.

## Supporting information


**Fig. S1**. Bioinformation analysis of genes upregulated by TGFβ1.
**Fig. S2**. Transforming growth factor β1 (TGFβ1) could upregulate laminin subunit gamma 1(LAMC1) expression at RNA level in a concentration‐ and time‐manner in esophageal squamous cell carcinoma (ESCC) cells.
**Fig. S3**. The details of predicted binding peaks of transcriptional factors SP1 and SMAD4 at the LAMC1 promoter region by hTFtarget.
**Fig. S4**. TGFβ1 upregulated the expression of transcriptional factors SP1 and SMAD4.
**Fig. S5**. Knockdown efficiency of SP1 combined with or without knockdown SMAD4in ESCC cells was verified by RT‐qPCR.
**Fig. S6**. Statistical column diagram of migration of these subgroups, shLAMC1 with or without TNFα (10 ng · mL^–1^) stimulation, overexpression LAMC1 with or without JSH‐23 stimulation, measured by chamber assay.
**Fig. S7**. TGFβ1 upregulated CXCL1 expression.
**Fig. S8**. Identification CAF and detection of the markers of iCAF or myCAF.
**Fig. S9**. Tumor‐secreted CXCL1 upregulated CXCR2 in CAF.
**Fig. S10**. Detection the expression of iCAF and myCAF markers in different CAF under PBS, IL8 or MIF treatments by RT‐qPCR.
**Fig. S11**. CM of tumor cells did not affect proliferation of CAF.
**Table S1**. Correlation analysis between the expression of LAMC1 and clinicopathologic parameters in ESCC patients.
**Table S2**. The predicted binding peaks of transcriptional factors SP1 and SMAD4 at the LAMC1 promoter region by hTFtarget.
**Table S3**. The predicted common targets for transcriptional factors SP1 and SMAD4 co‐regulation by hTFtarget.
**Table S4**. Primer sequences were used in our study.
**Table S5**. Antibodies were used for western blot in our study.
