# Supplementary material for: LAMC1 upregulation via TGFβ induces inflammatory cancer‐associated fibroblasts in esophageal squamous cell carcinoma via NF‐κB–CXCL1–STAT3
Source: Mol Oncol. 2021 Jul 22;15(11):3125–46. doi: 10.1002/1878-0261.13053 (PMC8564640; doi:10.1002/1878-0261.13053)
Supplement: Supplementary file 1 — Fig. S1. Bioinformation analysis of genes upregulated by TGFβ1. Fig. S2. Transforming growth factor β1 (TGFβ1) could upregulate laminin subunit gamma 1(LAMC1) expression at RNA level in a concentration‐ and time‐manner in esophageal squamous cell carcinoma (ESCC) cells. Fig. S3. The details of predicted binding peaks of transcriptional factors SP1 and SMAD4 at the LAMC1 promoter region by hTFtarget. Fig. S4. TGFβ1 upregulated the expression of transcriptional factors SP1 and SMAD4. Fig. S5. Knockdown efficiency of SP1 combined with or without knockdown SMAD4in ESCC cells was verified by RT‐qPCR. Fig. S6. Statistical column diagram of migration of these subgroups, shLAMC1 with or without TNFα (10 ng · mL–1) stimulation, overexpression LAMC1 with or without JSH‐23 stimulation, measured by chamber assay. Fig. S7. TGFβ1 upregulated CXCL1 expression. Fig. S8. Identification CAF and detection of the markers of iCAF or myCAF. Fig. S9. Tumor‐secreted CXCL1 upregulated CXCR2 in CAF. Fig. S10. Detection the expression of iCAF and myCAF markers in different CAF under PBS, IL8 or MIF treatments by RT‐qPCR. Fig. S11. CM of tumor cells did not affect proliferation of CAF. Table S1. Correlation analysis between the expression of LAMC1 and clinicopathologic parameters in ESCC patients. Table S2. The predicted binding peaks of transcriptional factors SP1 and SMAD4 at the LAMC1 promoter region by hTFtarget. Table S3. The predicted common targets for transcriptional factors SP1 and SMAD4 co‐regulation by hTFtarget. Table S4. Primer sequences were used in our study. Table S5. Antibodies were used for western blot in our study. [file MOL2-15-3125-s001.docx]

.  **Supplementary Material**

**Table S1. Correlation analysis between the expression of LAMC1 and clinicopathological parameters in ESCC patients.**

| **Variables** | **All cases** | **LAMC1** | | **χ**^2^ | ***P* ^ab^** |
| --- | --- | --- | --- | --- | --- |
|  |  | **Low-expression** | **High-expression** |  |  |
| **Tissue types** |  |  |  |  |  |
| Marginal | 49 | 41 | 8 | 23.916 | <0.001 |
| Tumor | 55 | 20 | 35 |  |  |
| **Stage** |  |  |  |  |  |
| stage 0-2 | 23 | 14 | 9 | 10.290 | 0.001 |
| stage 3-4 | 25 | 4 | 21 |  |  |
| **Age** |  |  |  |  |  |
| ≤60years | 33 | 13 | 20 | 0.327 | 0.567 |
| >60years | 22 | 7 | 15 |  |  |
| **Gender** |  |  |  |  |  |
| Male | 37 | 13 | 24 | 0.074 | 0.786 |
| Female | 18 | 7 | 11 |  |  |
| **Differentiation** |  |  |  |  |  |
| Ⅰ | 10 | 4 | 6 | 0.07 | 0.792 |
| Ⅱ+Ⅲ | 45 | 16 | 29 |  |  |
| **Distant metastasis** |  |  |  |  |  |
| yes | 50 | 19 | 31 | 0.636 | 0.425 |
| no | 5 | 1 | 4 |  |  |

^a^ *P* values for comparing clinicopathological parameters in LAMC1 low-expression

group verus high-expression group.

^b^ *P* <0.05 is considered significant.

^c^ missing 2cases

**Table S2.** **The predicted binding peaks of transcriptional factors SP1 and SMAD4 at the LAMC1 promoter region by hTFtarget.**

| **No. of datasets** | **TF** | **Tissue** | **No. of peaks (total/average)** | **No. of peaks in gene body (total/average)** | **No. of peaks around TSS (total/average)** | **The peak close to TSS** | **The peak with strongest signal** |
| --- | --- | --- | --- | --- | --- | --- | --- |
| 1 | SP1 | blood | 8/8 | 6/6 | 2/2 | chr1,183023205,183023318,4.88,-255,pr,dataset-3057 | chr1,183048770,183049002,24.7,2531,gb,  dataset-3057 |
| 2 | SP1 | colon | 12/6 | 9/4 | 3/1 | chr1,183023192,183024234,5.63,-268,pr,dataset-3066 | chr1,183022715,183023107,8.30,-745,pr,  dataset-3066 |
| 1 | SP1 | lung | 6/6 | 4/4 | 2/2 | chr1,183023219,183023407,3.41,-241,pr,dataset-3061 | chr1,183060469,183060826,8.68,3700,gb,  dataset-3061 |
| 1 | SMAD4 | embryo | 1/1 | 0 / 0 | 1/1 | chr1,183023168,183023327,5.24,-292,pr,dataset-2982 | chr1,183023168,183023327,5.24,-292,pr,dataset-2982 |

­­­­­­­

**Table S3. The predicted common targets for transcriptional factors SP1 and SMAD4 co-regulation by hTFtarget.**

| **TFs** | **Gene name** | **Chromosome** | **Start** | **End** |
| --- | --- | --- | --- | --- |
| SMAD4SP1 | DDX20 | chr1 | 111755245 | 111768016 |
| SMAD4SP1 | RAP1GAP | chr1 | 21596215 | 21669363 |
| SMAD4SP1 | MAST2 | chr1 | 45786987 | 46036124 |
| SMAD4SP1 | C1orf216 | chr1 | 35713875 | 35719472 |
| SMAD4SP1 | MIR6735 | chr1 | 43448539 | 43448611 |
| SMAD4SP1 | KDF1 | chr1 | 26949562 | 26960406 |
| SMAD4SP1 | KLHL17 | chr1 | 960587 | 965715 |
| SMAD4SP1 | WDR64 | chr1 | 241652278 | 241802133 |
| SMAD4SP1 | LAMC1 | chr1 | 183023460 | 183145592 |
| SMAD4SP1 | RP11-359K18.3 | chr1 | 162560227 | 162561308 |
| SMAD4SP1 | TRIM46 | chr1 | 155173787 | 155184971 |
| SMAD4SP1 | KRTCAP2 | chr1 | 155169408 | 155173475 |
| SMAD4SP1 | NFIA | chr1 | 60865259 | 61462793 |
| SMAD4SP1 | MEGF6 | chr1 | 3489920 | 3611495 |
| SMAD4SP1 | POLR3C | chr1 | 145824088 | 145842505 |
| SMAD4SP1 | MIR6740 | chr1 | 202003124 | 202003236 |
| SMAD4SP1 | MIR548AC | chr1 | 116560024 | 116560111 |
| SMAD4SP1 | MIR30C1 | chr1 | 40757284 | 40757372 |
| SMAD4SP1 | RP4-639F20.1 | chr1 | 94927566 | 94963270 |
| SMAD4SP1 | SNORA26 | chr1 | 156192063 | 156192203 |
| SMAD4SP1 | RP11-131M11.3 | chr1 | 33162851 | 33166298 |
| SMAD4SP1 | PHF13 | chr1 | 6613685 | 6624033 |
| SMAD4SP1 | RHOU | chr1 | 228735077 | 228746669 |

**Table S4. Primer sequences were used in our study**

| **Gene** | **Primer sequence** | |
| --- | --- | --- |
|  | **Forward** | **Reverse** |
| **β_Actin** | CTACCTCATGAAGATCCTCACCGA | TTCTCCTTAATGTCACGCACGATT |
| **LAMC1** | ATTTCAATCAACCGCTCT | GTTATGGACCTCCTTCGT |
| **SP1** | GGCTGGTGGTGATGGAATAC | GCCCCTTCCTTCACTGTCTT |
| **SMAD4** | TCCACTTGAATGCTGCTCT | GTTGTTTGCTGGTGTCCTC |
| **IL1β** | GCCCAAGATGAAGACCAACCAGT | CCGTGAGTTTCCCAGAAGAAGAGG |
| **IL6** | CCTCCAGAACAGATTTGAGAGTAGT | GGGTCAGGGGTGGTTATTGC |
| **LIF** | TGAGGGCACTGGGGTTGAGGA | AGAAGGCCAAGCTGGTGGAGC |
| **CXCL1** | TCACATCTAACCTCATCTTCTTCAC | ACTCTTCACATAGCACATTGTTCTC |
| **CSF3** | CCTGCATTTCTGAGTTTCATTCT | GCTGGGGAGCAGTCATAGTA |
| **ACTA2** | CCTTGAGAAGAGTTACGAGTTGC | ATGATGCTGTTGTAGGTGGTTT |
| **CTGF** | CCTGTCTTACTTTTCCGAAGGAC | CGTCAGGGCACTTGAACTCC |
| **CXCR2** | TGGATGCAGAACTTGACAACGT | TTGCTGTATTGTTGCCCATGT |
| **ChIP-primer** | AGCTCAGCCGTGTAACAAGA | CTGGATACGGCAAGCAGAGA |

**Table S5.** **Antibodies were used for western blot in our study**

| **Antibodies** | **Catalog no.** | **Company** |
| --- | --- | --- |
| **Anti-β_Actin-antibody** | A1978 | **Sigma Aldrich** |
| **Anti-LAMC1-antibody** | **AP20488PU-N** | **OriGene** |
| **Anti-SP1-antibody** | 9389 | **Cell Signaling Technology** |
| **Anti-SMAD4-antibody** | 46535 | **Cell Signaling Technology** |
| **Anti-Akt-antibody** | 4691 | **Cell Signaling Technology** |
| **Anti-p-Akt-antibody** | 4060 | **Cell Signaling Technology** |
| **Anti-p65(Ser536)-antibody** | 8242 | **Cell Signaling Technology** |
| **Anti-p-p65(Ser536)-antibody** | ab76302 | **Abcam** |
| **Anti-IKKα-antibody** | 61294 | **Cell Signaling Technology** |
| **Anti-pIKKα-antibody** | AP0505 | **ABclonal** |
| **Anti-MMP9-antibody** | 13667 | **Cell Signaling Technology** |
| **Anti-MMP14-antibody** | Ab53712 | **Abcam** |
| **Anti-cleaved caspase3-antibody** | 9664S | **Cell Signaling Technology** |
| **Anti- cleaved caspase9-antibody** | 7237 | **Cell Signaling Technology** |
| **Anti- cleaved PARP-antibody** | 5625S | **Cell Signaling Technology** |
| **Anti-CXCL1-antibody** | ab206411 | **Abcam** |
| **Anti-IL8-antibody** | A2541 | **ABclonal** |
| **Anti-MIF-antibody** | A11231 | **ABclonal** |
| **Anti-αSMA-antibody** | 8456 | **Cell Signaling Technology** |
| **Anti-IL6-antibody** | 12153 | **Cell Signaling Technology** |
| **Anti-CSF3-antibody** | A6178 | **ABclonal** |
| **Anti-IL1β -antibody** | 83186 | **Cell Signaling Technology** |
| **Anti-STAT3-antibody** | 30385 | **Cell Signaling Technology** |
| **Anti-pSTAT3-antibody** | 9145 | **Cell Signaling Technology** |
| **Anti-CXCR2-antibody** | ab14935 | **Abcam** |
| **Anti-rabbit IgG, HRP-linked antibody** | 7074 | **Cell Signaling Technology** |
| **Anti-mouse IgG, HRP-linked antibody** | 7076 | **Cell Signaling Technology** |


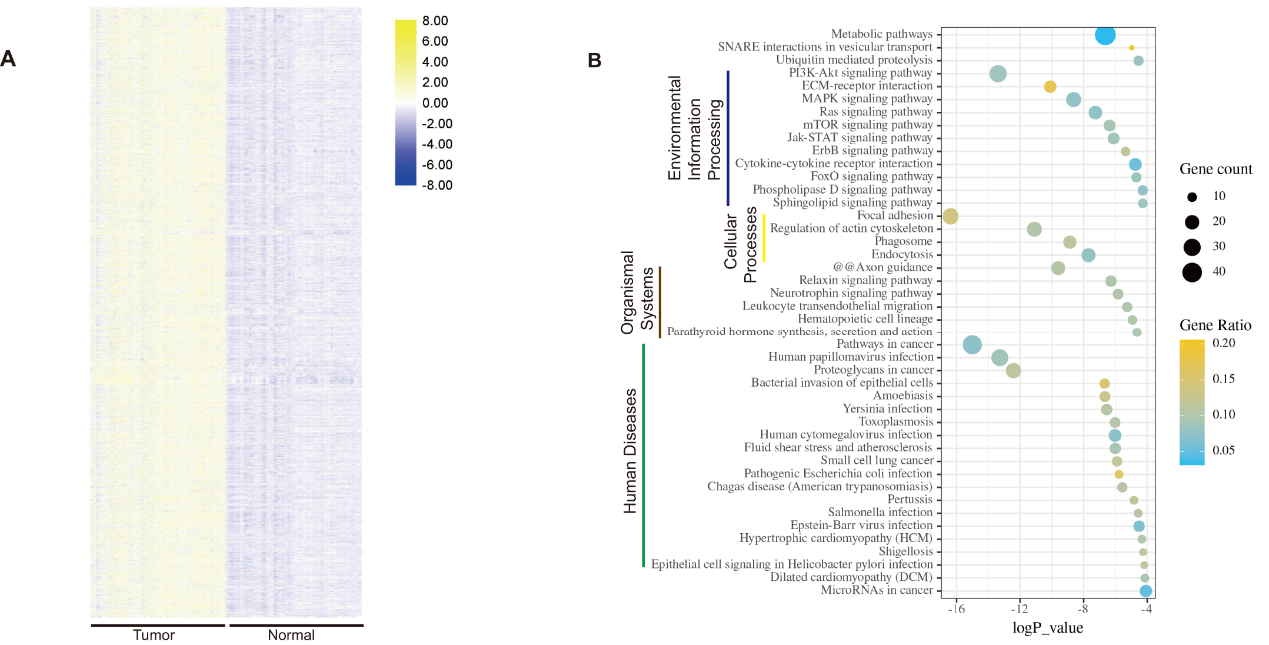


Fig S1. Bioinformation analysis of genes upregulated by TGFβ1. (A) Heatmap of 4130 genes upregulated in tumors compared with para-cancer tissues (GSE53625). (B) The enriched KEGG pathways of the 652 genes upregulated by Transforming growth factor β1 (TGFβ1) (p<0.0001).


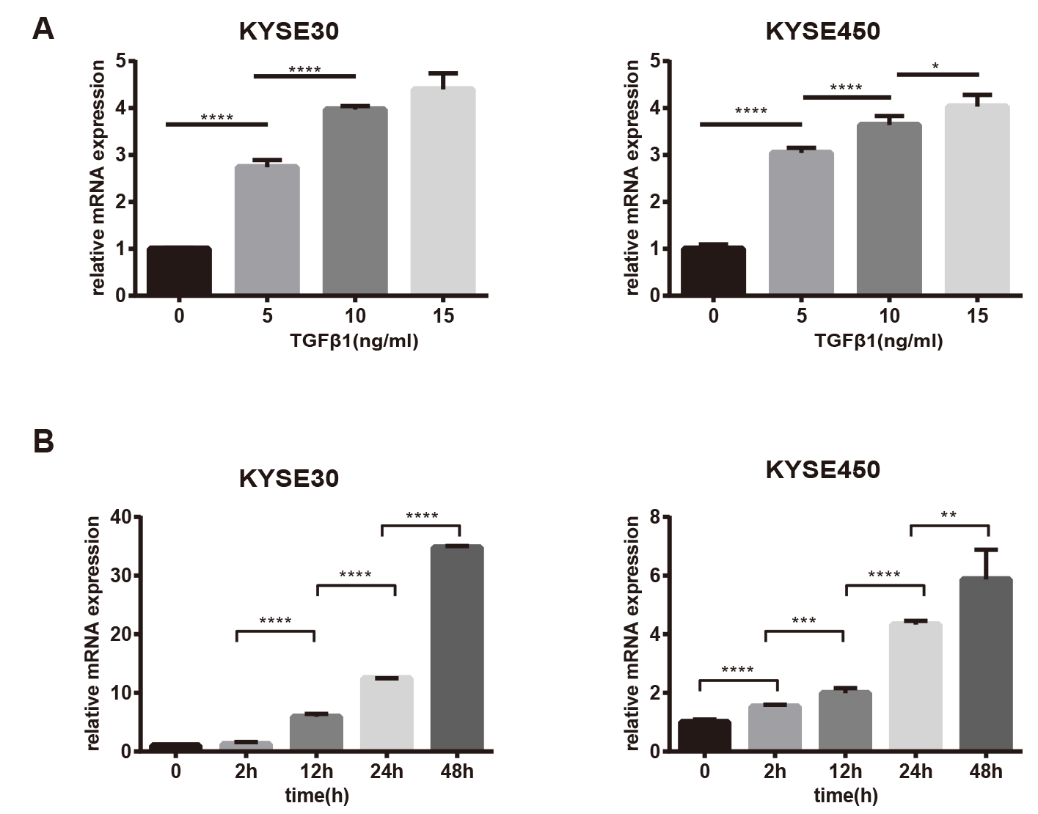


Fig S2. Transforming growth factor β1 (TGFβ1) could upregulate laminin subunit gamma 1(LAMC1) expression at RNA level in a concentration- and time- manner in esophageal squamous cell carcinoma (ESCC) cells. (A-B) TGFβ1 incubated KYSE30 and KYSE450 cells were treated with different concentration for 24h (A) or at 5 ng/ml for different times (B), the expression levels of LAMC1 were measured by RT-qPCR. Three biological replicates were performed for *in vitro* assays. Data in bar charts are presented as the mean ± SD. **p < 0.01, ***p < 0.001, ****p < 0.0001 (Student’s t test).


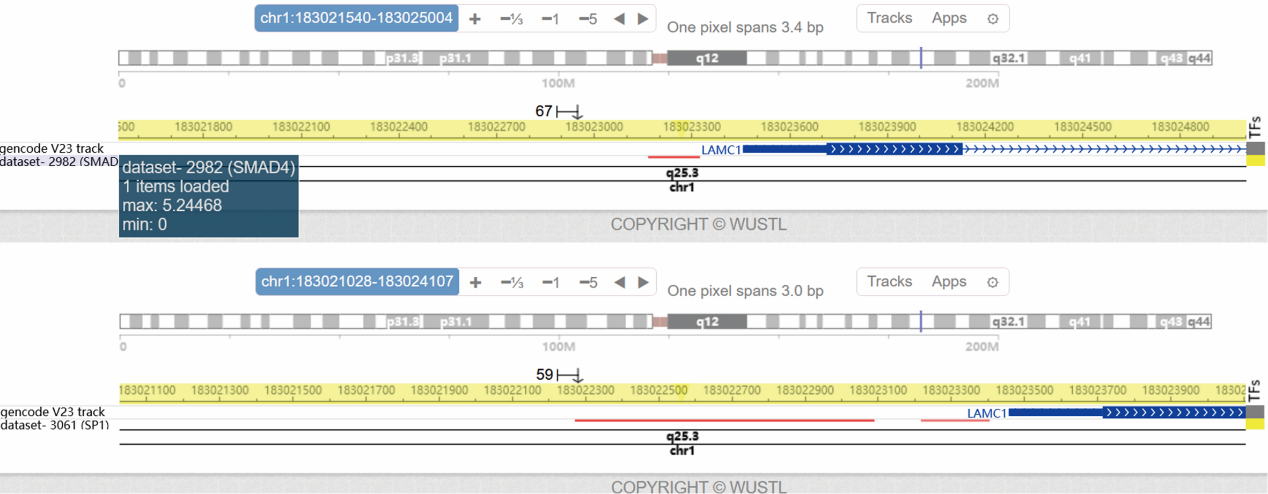


Fig S3. The details of predicted binding peaks of transcriptional factors SP1 and SMAD4 at the LAMC1 promoter region by hTFtarget.


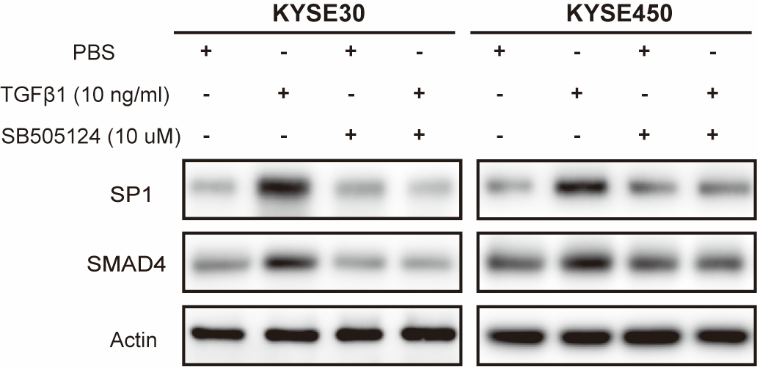


Fig S4. TGFβ1 upregulated the expression of transcriptional factors SP1 and SMAD4.

The expression of SMAD4 and SP1 were upregulated in KYSE30 and KYSE450 cells after TGFβ1 treatments, which could be reversed by TGFβ receptor inhibitor SB505124. Three biological replicates were performed for *in vitro* assays.


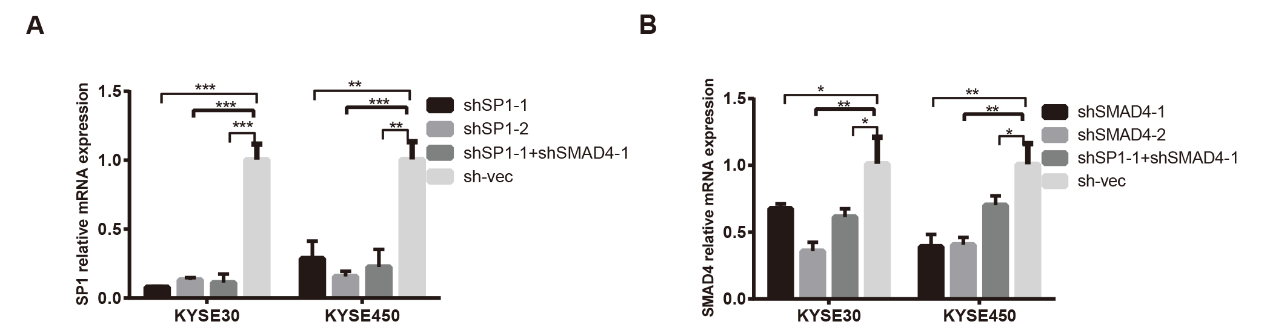


Fig S5. (A-B) Knockdown efficiency of SP1 (A) combined with or without knockdown SMAD4 (B) in ESCC cells was verified by RT-qPCR. Three biological replicates were performed for *in vitro* assays. Data in bar charts are presented as the mean ± SD. *p < 0.05, **p < 0.01, ***p < 0.001, ****p < 0.0001 (Student’s t test).


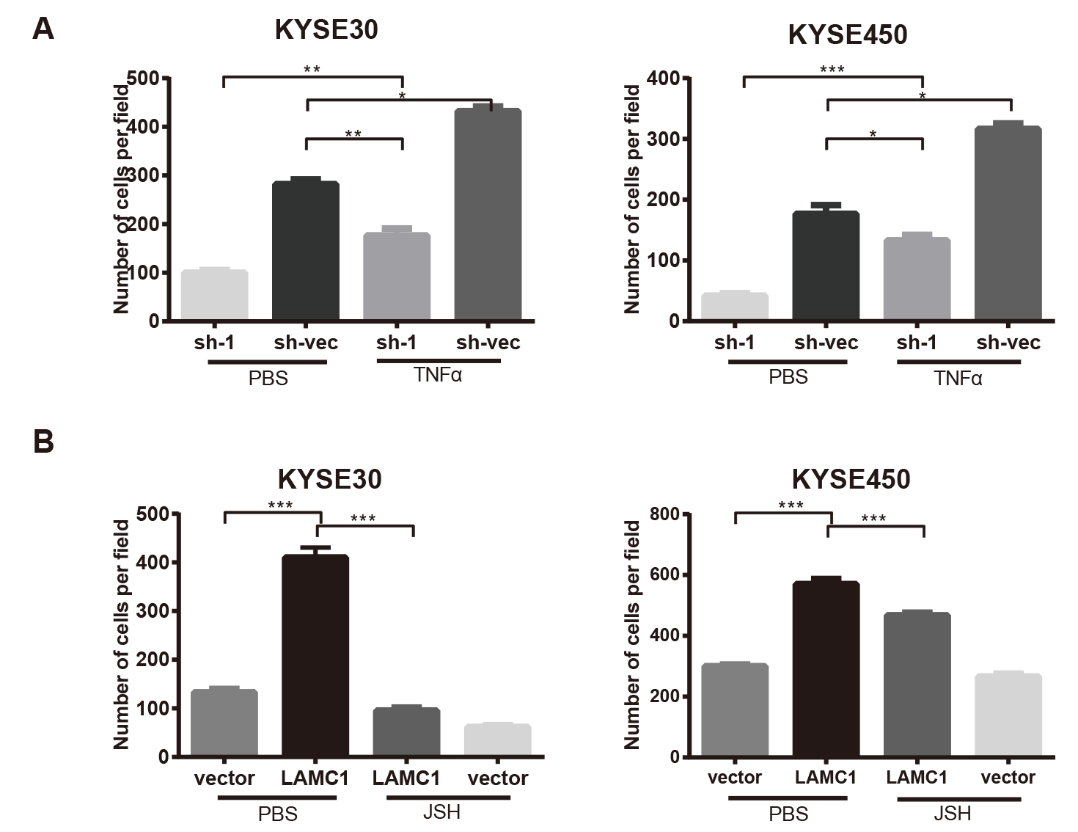


Fig S6. (A-B) Statistical column diagram of migration of these subgroups, shLAMC1 with or without TNFα (10 ng/ml) stimulation, overexpression LAMC1 with or without JSH-23 stimulation, measured by chamber assay. Three biological replicates were performed for *in vitro* assays. Data in bar charts are presented as the mean ± SD. *p < 0.05, **p < 0.01, ***p < 0.001 (Student’s t test).


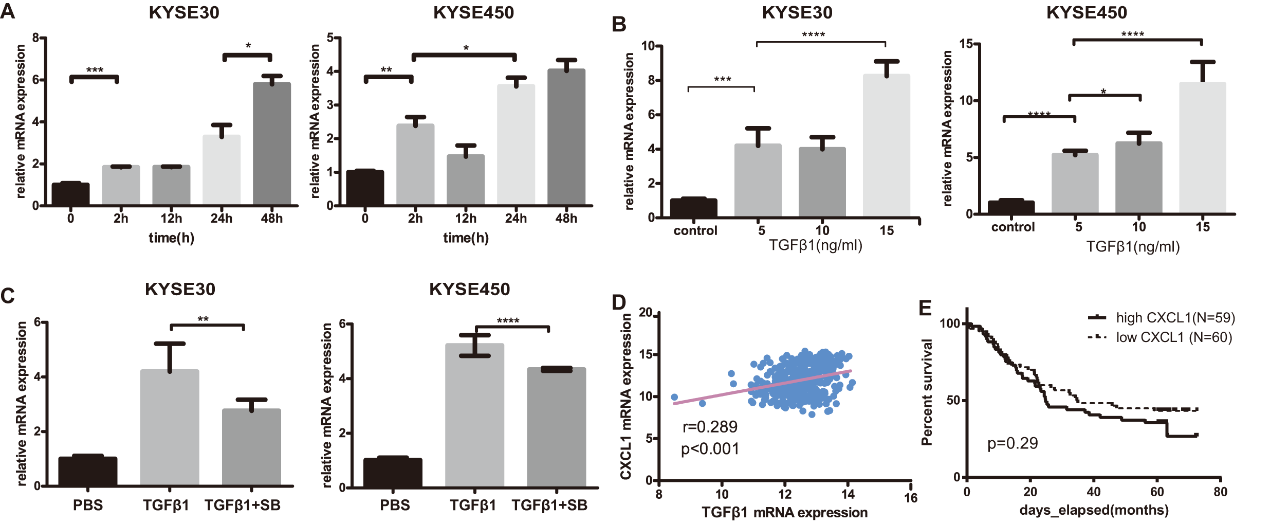


Fig S7. TGFβ1 upregulated CXCL1 expression. (A-C) By RT-qPCR, TGFβ1 upregulated CXCL1 expression at the protein and RNA levels in a concentration- and time-dependent manner in KYSE30 and KYSE450 cells, which could be reversed by SB505124. (D) TGFβ1 was positively associated with CXCL1 based on GSE53625 data. (E) Expression of CXCL1 was not associated with poor prognosis based on GSE53625. Three biological replicates were performed for in vitro assays. Data in bar charts are presented as the mean ± SD. *p < 0.05, **p < 0.01, ***p < 0.001, ****p < 0.0001 (Student’s t test).


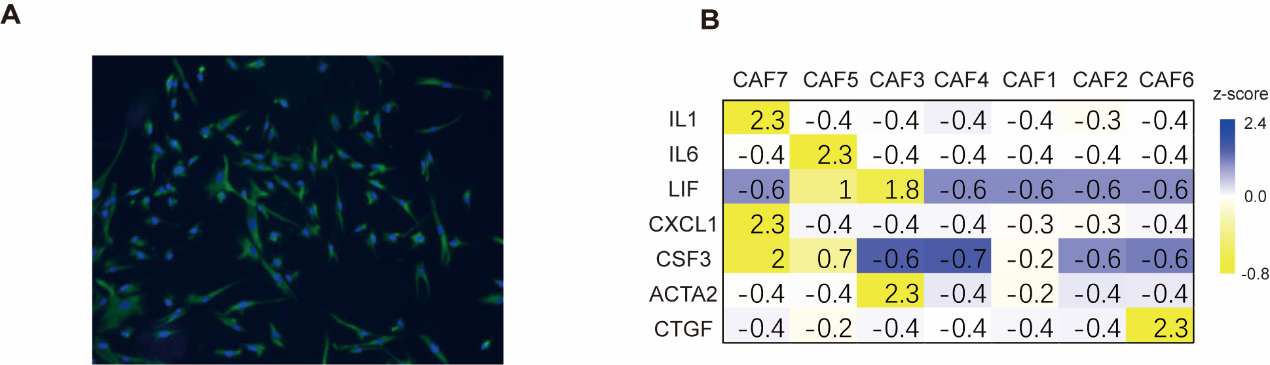


Fig S8. Identification CAFs and detection the markers of iCAFs or myCAFs. (A) Immunofluorescence showing the high expression of α-SMA in isolated CAFs. (B) The expression of the markers of iCAFs or myCAF in 7 different CAFs derived from different ESCC patients’ samples was detected by RT-qPCR (relative to CAF1). Three biological replicates were performed for in vitro assays.

**
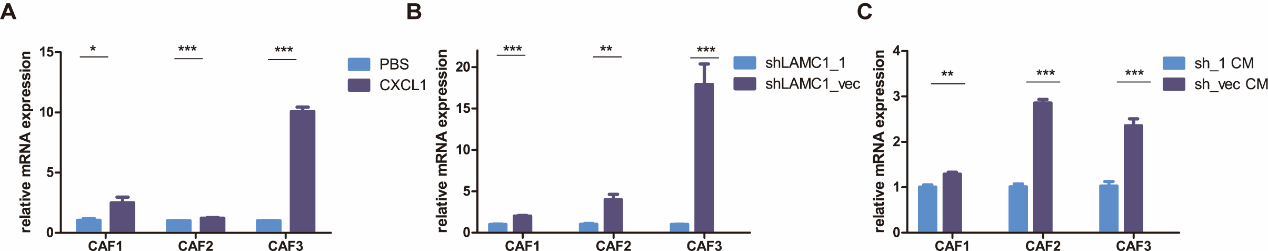
**

Fig S9. Tumor-secreted CXCL1 upregulated CXCR2 in CAFs. Expression of CXCR2 was detected in CAFs with three different treatments: induced with rCXCL1 (A), cocultured with shLAMC1 and sh-vec KYSE30 cells (B), or stimulated with CM from these tumor cells (C), as demonstrated by RT-qPCR. Three biological replicates were performed for in vitro assays. Data in bar charts are presented as the mean ± SD. *p < 0.05, **p < 0.01, ***p < 0.001, ****p < 0.0001 (Student’s t test).


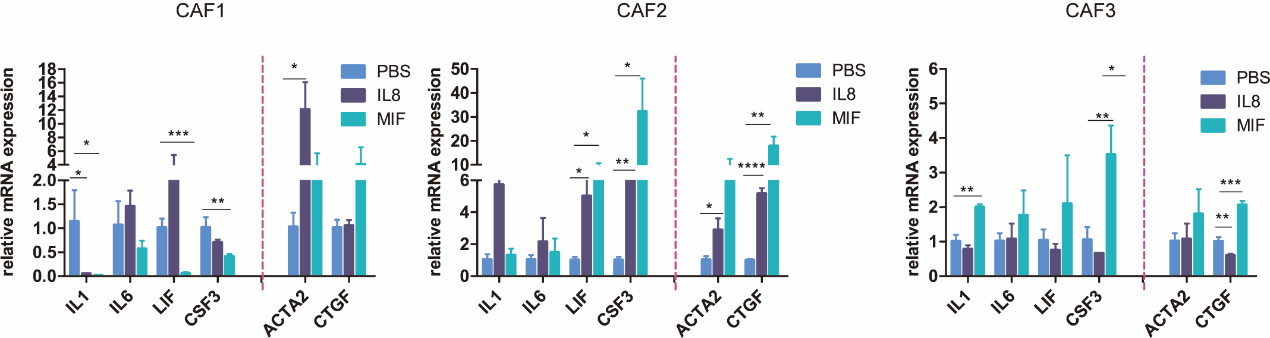


Fig S10. Detection the expression of iCAF and myCAF markers in different CAFs under PBS, IL8 or MIF treatments by RT-qPCR. Three biological replicates were performed for in vitro assays. Data in bar charts are presented as the mean ± SD. *p < 0.05, **p < 0.01, ***p < 0.001, ****p < 0.0001 (Student’s t test).


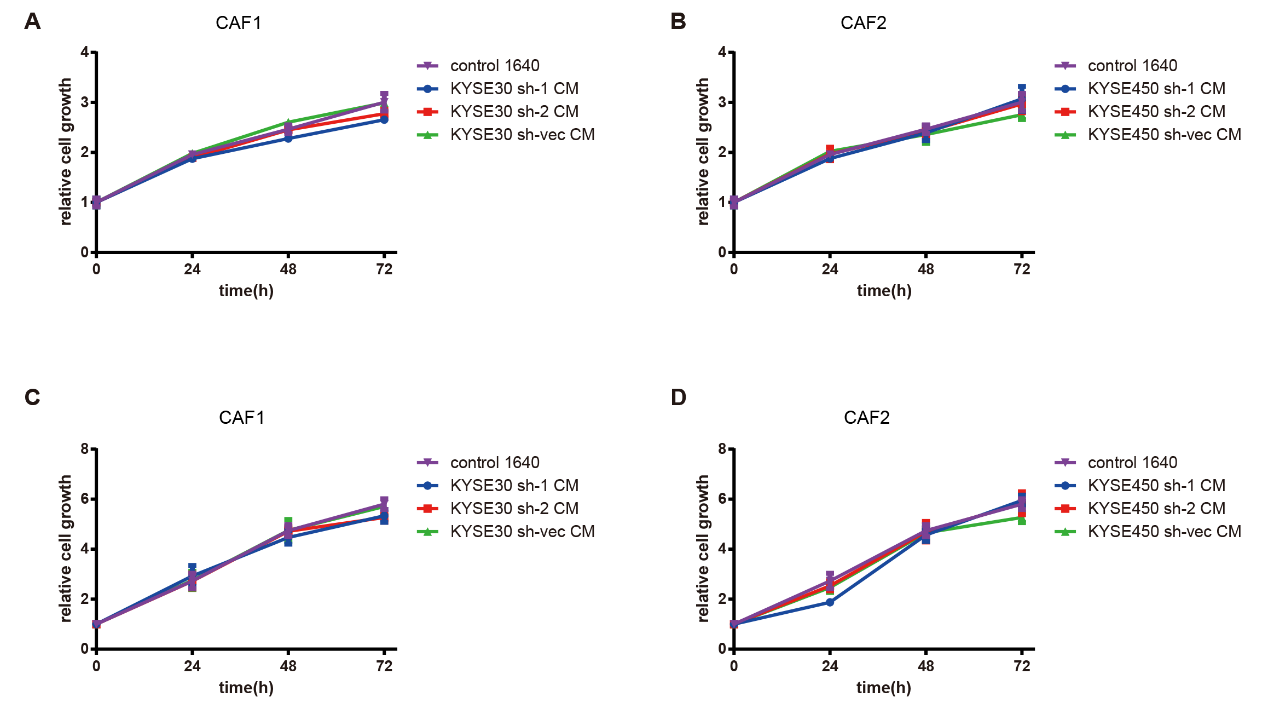


Fig S11. CM of tumor cells did not affect proliferation of CAFs. (A-D）Proliferation of CAFs treated with CM of tumor cells measured by CCK8 assay. Three biological replicates were performed for in vitro assays. Data in bar charts are presented as the mean ± SD.
